# Studies of ${\mathrm {B}} ^{*}_{{\mathrm {s}}2}(5840)^0$ and ${\mathrm {B}} _{{\mathrm {s}}1}(5830)^0$ mesons including the observation of the ${\mathrm {B}} ^{*}_{{\mathrm {s}}2}(5840)^0 \rightarrow {\mathrm {B}} ^0 \mathrm {K} ^0_{\mathrm {S}}$ decay in proton-proton collisions at $\sqrt{s}=8\,\text {TeV}$

**DOI:** 10.1140/epjc/s10052-018-6390-z

**Published:** 2018-11-15

**Authors:** A. M. Sirunyan, A. Tumasyan, W. Adam, F. Ambrogi, E. Asilar, T. Bergauer, J. Brandstetter, M. Dragicevic, J. Erö, A. Escalante Del Valle, M. Flechl, R. Frühwirth, V. M. Ghete, J. Hrubec, M. Jeitler, N. Krammer, I. Krätschmer, D. Liko, T. Madlener, I. Mikulec, N. Rad, H. Rohringer, J. Schieck, R. Schöfbeck, M. Spanring, D. Spitzbart, A. Taurok, W. Waltenberger, J. Wittmann, C.-E. Wulz, M. Zarucki, V. Chekhovsky, V. Mossolov, J. Suarez Gonzalez, E. A. De Wolf, D. Di Croce, X. Janssen, J. Lauwers, M. Pieters, H. Van Haevermaet, P. Van Mechelen, N. Van Remortel, S. Abu Zeid, F. Blekman, J. D’Hondt, I. De Bruyn, J. De Clercq, K. Deroover, G. Flouris, D. Lontkovskyi, S. Lowette, I. Marchesini, S. Moortgat, L. Moreels, Q. Python, K. Skovpen, S. Tavernier, W. Van Doninck, P. Van Mulders, I. Van Parijs, D. Beghin, B. Bilin, H. Brun, B. Clerbaux, G. De Lentdecker, H. Delannoy, B. Dorney, G. Fasanella, L. Favart, R. Goldouzian, A. Grebenyuk, A. K. Kalsi, T. Lenzi, J. Luetic, N. Postiau, E. Starling, L. Thomas, C. Vander Velde, P. Vanlaer, D. Vannerom, Q. Wang, T. Cornelis, D. Dobur, A. Fagot, M. Gul, I. Khvastunov, D. Poyraz, C. Roskas, D. Trocino, M. Tytgat, W. Verbeke, B. Vermassen, M. Vit, N. Zaganidis, H. Bakhshiansohi, O. Bondu, S. Brochet, G. Bruno, C. Caputo, P. David, C. Delaere, M. Delcourt, A. Giammanco, G. Krintiras, V. Lemaitre, A. Magitteri, A. Mertens, M. Musich, K. Piotrzkowski, A. Saggio, M. Vidal Marono, S. Wertz, J. Zobec, F. L. Alves, G. A. Alves, M. Correa Martins Junior, G. Correia Silva, C. Hensel, A. Moraes, M. E. Pol, P. Rebello Teles, E. Belchior Batista Das Chagas, W. Carvalho, J. Chinellato, E. Coelho, E. M. Da Costa, G. G. Da Silveira, D. De Jesus Damiao, C. De Oliveira Martins, S. Fonseca De Souza, H. Malbouisson, D. Matos Figueiredo, M. Melo De Almeida, C. Mora Herrera, L. Mundim, H. Nogima, W. L. Prado Da Silva, L. J. Sanchez Rosas, A. Santoro, A. Sznajder, M. Thiel, E. J. Tonelli Manganote, F. Torres Da Silva De Araujo, A. Vilela Pereira, S. Ahuja, C. A. Bernardes, L. Calligaris, T. R. Fernandez Perez Tomei, E. M. Gregores, P. G. Mercadante, S. F. Novaes, Sandra S. Padula, A. Aleksandrov, R. Hadjiiska, P. Iaydjiev, A. Marinov, M. Misheva, M. Rodozov, M. Shopova, G. Sultanov, A. Dimitrov, L. Litov, B. Pavlov, P. Petkov, W. Fang, X. Gao, L. Yuan, M. Ahmad, J. G. Bian, G. M. Chen, H. S. Chen, M. Chen, Y. Chen, C. H. Jiang, D. Leggat, H. Liao, Z. Liu, F. Romeo, S. M. Shaheen, A. Spiezia, J. Tao, Z. Wang, E. Yazgan, H. Zhang, S. Zhang, J. Zhao, Y. Ban, G. Chen, A. Levin, J. Li, L. Li, Q. Li, Y. Mao, S. J. Qian, D. Wang, Z. Xu, Y. Wang, C. Avila, A. Cabrera, C. A. Carrillo Montoya, L. F. Chaparro Sierra, C. Florez, C. F. González Hernández, M. A. Segura Delgado, B. Courbon, N. Godinovic, D. Lelas, I. Puljak, T. Sculac, Z. Antunovic, M. Kovac, V. Brigljevic, D. Ferencek, K. Kadija, B. Mesic, A. Starodumov, T. Susa, M. W. Ather, A. Attikis, M. Kolosova, G. Mavromanolakis, J. Mousa, C. Nicolaou, F. Ptochos, P. A. Razis, H. Rykaczewski, M. Finger, M. Finger, E. Ayala, E. Carrera Jarrin, A. Mahrous, A. Mohamed, E. Salama, S. Bhowmik, A. Carvalho Antunes De Oliveira, R. K. Dewanjee, K. Ehataht, M. Kadastik, M. Raidal, C. Veelken, P. Eerola, H. Kirschenmann, J. Pekkanen, M. Voutilainen, J. Havukainen, J. K. Heikkilä, T. Järvinen, V. Karimäki, R. Kinnunen, T. Lampén, K. Lassila-Perini, S. Laurila, S. Lehti, T. Lindén, P. Luukka, T. Mäenpää, H. Siikonen, E. Tuominen, J. Tuominiemi, T. Tuuva, M. Besancon, F. Couderc, M. Dejardin, D. Denegri, J. L. Faure, F. Ferri, S. Ganjour, A. Givernaud, P. Gras, G. Hamel de Monchenault, P. Jarry, C. Leloup, E. Locci, J. Malcles, G. Negro, J. Rander, A. Rosowsky, M. Ö. Sahin, M. Titov, A. Abdulsalam, C. Amendola, I. Antropov, F. Beaudette, P. Busson, C. Charlot, R. Granier de Cassagnac, I. Kucher, A. Lobanov, J. Martin Blanco, C. Martin Perez, M. Nguyen, C. Ochando, G. Ortona, P. Paganini, P. Pigard, J. Rembser, R. Salerno, J. B. Sauvan, Y. Sirois, A. G. Stahl Leiton, A. Zabi, A. Zghiche, J.-L. Agram, J. Andrea, D. Bloch, J.-M. Brom, E. C. Chabert, V Cherepanov, C. Collard, E. Conte, J.-C. Fontaine, D. Gelé, U. Goerlach, M. Jansová, A.-C. Le Bihan, N. Tonon, P. Van Hove, S. Gadrat, S. Beauceron, C. Bernet, G. Boudoul, N. Chanon, R. Chierici, D. Contardo, P. Depasse, H. El Mamouni, J. Fay, L. Finco, S. Gascon, M. Gouzevitch, G. Grenier, B. Ille, F. Lagarde, I. B. Laktineh, H. Lattaud, M. Lethuillier, L. Mirabito, S. Perries, A. Popov, V. Sordini, G. Touquet, M. Vander Donckt, S. Viret, T. Toriashvili, D. Lomidze, C. Autermann, L. Feld, M. K. Kiesel, K. Klein, M. Lipinski, M. Preuten, M. P. Rauch, C. Schomakers, J. Schulz, M. Teroerde, B. Wittmer, A. Albert, D. Duchardt, M. Erdmann, S. Erdweg, T. Esch, R. Fischer, S. Ghosh, A. Güth, T. Hebbeker, C. Heidemann, K. Hoepfner, H. Keller, L. Mastrolorenzo, M. Merschmeyer, A. Meyer, P. Millet, S. Mukherjee, T. Pook, M. Radziej, H. Reithler, M. Rieger, A. Schmidt, D. Teyssier, S. Thüer, G. Flügge, O. Hlushchenko, T. Kress, A. Künsken, T. Müller, A. Nehrkorn, A. Nowack, C. Pistone, O. Pooth, D. Roy, H. Sert, A. Stahl, M. Aldaya Martin, T. Arndt, C. Asawatangtrakuldee, I. Babounikau, K. Beernaert, O. Behnke, U. Behrens, A. Bermúdez Martínez, D. Bertsche, A. A. Bin Anuar, K. Borras, V. Botta, A. Campbell, P. Connor, C. Contreras-Campana, V. Danilov, A. De Wit, M. M. Defranchis, C. Diez Pardos, D. Domínguez Damiani, G. Eckerlin, T. Eichhorn, A. Elwood, E. Eren, E. Gallo, A. Geiser, A. Grohsjean, M. Guthoff, M. Haranko, A. Harb, J. Hauk, H. Jung, M. Kasemann, J. Keaveney, C. Kleinwort, J. Knolle, D. Krücker, W. Lange, A. Lelek, T. Lenz, J. Leonard, K. Lipka, W. Lohmann, R. Mankel, I.-A. Melzer-Pellmann, A. B. Meyer, M. Meyer, M. Missiroli, G. Mittag, J. Mnich, V. Myronenko, S. K. Pflitsch, D. Pitzl, A. Raspereza, M. Savitskyi, P. Saxena, P. Schütze, C. Schwanenberger, R. Shevchenko, A. Singh, H. Tholen, O. Turkot, A. Vagnerini, G. P. Van Onsem, R. Walsh, Y. Wen, K. Wichmann, C. Wissing, O. Zenaiev, R. Aggleton, S. Bein, L. Benato, A. Benecke, V. Blobel, T. Dreyer, A. Ebrahimi, E. Garutti, D. Gonzalez, P. Gunnellini, J. Haller, A. Hinzmann, A. Karavdina, G. Kasieczka, R. Klanner, R. Kogler, N. Kovalchuk, S. Kurz, V. Kutzner, J. Lange, D. Marconi, J. Multhaup, M. Niedziela, C. E. N. Niemeyer, D. Nowatschin, A. Perieanu, A. Reimers, O. Rieger, C. Scharf, P. Schleper, S. Schumann, J. Schwandt, J. Sonneveld, H. Stadie, G. Steinbrück, F. M. Stober, M. Stöver, A. Vanhoefer, B. Vormwald, I. Zoi, M. Akbiyik, C. Barth, M. Baselga, S. Baur, E. Butz, R. Caspart, T. Chwalek, F. Colombo, W. De Boer, A. Dierlamm, K. El Morabit, N. Faltermann, B. Freund, M. Giffels, M. A. Harrendorf, F. Hartmann, S. M. Heindl, U. Husemann, F. Kassel, I. Katkov, S. Kudella, S. Mitra, M. U. Mozer, Th. Müller, M. Plagge, G. Quast, K. Rabbertz, M. Schröder, I. Shvetsov, G. Sieber, H. J. Simonis, R. Ulrich, S. Wayand, M. Weber, T. Weiler, S. Williamson, C. Wöhrmann, R. Wolf, G. Anagnostou, G. Daskalakis, T. Geralis, A. Kyriakis, D. Loukas, G. Paspalaki, I. Topsis-Giotis, G. Karathanasis, S. Kesisoglou, P. Kontaxakis, A. Panagiotou, I. Papavergou, N. Saoulidou, E. Tziaferi, K. Vellidis, K. Kousouris, I. Papakrivopoulos, G. Tsipolitis, I. Evangelou, C. Foudas, P. Gianneios, P. Katsoulis, P. Kokkas, S. Mallios, N. Manthos, I. Papadopoulos, E. Paradas, J. Strologas, F. A. Triantis, D. Tsitsonis, M. Bartók, M. Csanad, N. Filipovic, P. Major, M. I. Nagy, G. Pasztor, O. Surányi, G. I. Veres, G. Bencze, C. Hajdu, D. Horvath, Á. Hunyadi, F. Sikler, T. Á. Vámi, V. Veszpremi, G. Vesztergombi, N. Beni, S. Czellar, J. Karancsi, A. Makovec, J. Molnar, Z. Szillasi, P. Raics, Z. L. Trocsanyi, B. Ujvari, S. Choudhury, J. R. Komaragiri, P. C. Tiwari, S. Bahinipati, C. Kar, P. Mal, K. Mandal, A. Nayak, D. K. Sahoo, S. K. Swain, S. Bansal, S. B. Beri, V. Bhatnagar, S. Chauhan, R. Chawla, N. Dhingra, R. Gupta, A. Kaur, M. Kaur, S. Kaur, R. Kumar, P. Kumari, M. Lohan, A. Mehta, K. Sandeep, S. Sharma, J. B. Singh, A. K. Virdi, G. Walia, A. Bhardwaj, B. C. Choudhary, R. B. Garg, M. Gola, S. Keshri, Ashok Kumar, S. Malhotra, M. Naimuddin, P. Priyanka, K. Ranjan, Aashaq Shah, R. Sharma, R. Bhardwaj, M. Bharti, R. Bhattacharya, S. Bhattacharya, U. Bhawandeep, D. Bhowmik, S. Dey, S. Dutt, S. Dutta, S. Ghosh, K. Mondal, S. Nandan, A. Purohit, P. K. Rout, A. Roy, S. Roy Chowdhury, G. Saha, S. Sarkar, M. Sharan, B. Singh, S. Thakur, P. K. Behera, R. Chudasama, D. Dutta, V. Jha, V. Kumar, P. K. Netrakanti, L. M. Pant, P. Shukla, T. Aziz, M. A. Bhat, S. Dugad, G. B. Mohanty, N. Sur, B. Sutar, RavindraKumar Verma, S. Banerjee, S. Bhattacharya, S. Chatterjee, P. Das, M. Guchait, Sa. Jain, S. Karmakar, S. Kumar, M. Maity, G. Majumder, K. Mazumdar, N. Sahoo, T. Sarkar, S. Chauhan, S. Dube, V. Hegde, A. Kapoor, K. Kothekar, S. Pandey, A. Rane, S. Sharma, S. Chenarani, E. Eskandari Tadavani, S. M. Etesami, M. Khakzad, M. Mohammadi Najafabadi, M. Naseri, F. Rezaei Hosseinabadi, B. Safarzadeh, M. Zeinali, M. Felcini, M. Grunewald, M. Abbrescia, C. Calabria, A. Colaleo, D. Creanza, L. Cristella, N. De Filippis, M. De Palma, A. Di Florio, F. Errico, L. Fiore, A. Gelmi, G. Iaselli, M. Ince, S. Lezki, G. Maggi, M. Maggi, G. Miniello, S. My, S. Nuzzo, A. Pompili, G. Pugliese, R. Radogna, A. Ranieri, G. Selvaggi, A. Sharma, L. Silvestris, R. Venditti, P. Verwilligen, G. Zito, G. Abbiendi, C. Battilana, D. Bonacorsi, L. Borgonovi, S. Braibant-Giacomelli, R. Campanini, P. Capiluppi, A. Castro, F. R. Cavallo, S. S. Chhibra, C. Ciocca, G. Codispoti, M. Cuffiani, G. M. Dallavalle, F. Fabbri, A. Fanfani, E. Fontanesi, P. Giacomelli, C. Grandi, L. Guiducci, S. Lo Meo, S. Marcellini, G. Masetti, A. Montanari, F. L. Navarria, A. Perrotta, F. Primavera, A. M. Rossi, T. Rovelli, G. P. Siroli, N. Tosi, S. Albergo, A. Di Mattia, R. Potenza, A. Tricomi, C. Tuve, G. Barbagli, K. Chatterjee, V. Ciulli, C. Civinini, R. D’Alessandro, E. Focardi, G. Latino, P. Lenzi, M. Meschini, S. Paoletti, L. Russo, G. Sguazzoni, D. Strom, L. Viliani, L. Benussi, S. Bianco, F. Fabbri, D. Piccolo, F. Ferro, F. Ravera, E. Robutti, S. Tosi, A. Benaglia, A. Beschi, F. Brivio, V. Ciriolo, S. Di Guida, M. E. Dinardo, S. Fiorendi, S. Gennai, A. Ghezzi, P. Govoni, M. Malberti, S. Malvezzi, A. Massironi, D. Menasce, F. Monti, L. Moroni, M. Paganoni, D. Pedrini, S. Ragazzi, T. Tabarelli de Fatis, D. Zuolo, S. Buontempo, N. Cavallo, A. De Iorio, A. Di Crescenzo, F. Fabozzi, F. Fienga, G. Galati, A. O. M. Iorio, W. A. Khan, L. Lista, S. Meola, P. Paolucci, C. Sciacca, E. Voevodina, P. Azzi, N. Bacchetta, D. Bisello, A. Boletti, A. Bragagnolo, R. Carlin, P. Checchia, M. Dall’Osso, P. De Castro Manzano, T. Dorigo, U. Dosselli, F. Gasparini, U. Gasparini, A. Gozzelino, S. Y. Hoh, S. Lacaprara, P. Lujan, M. Margoni, A. T. Meneguzzo, J. Pazzini, P. Ronchese, R. Rossin, F. Simonetto, A. Tiko, E. Torassa, M. Zanetti, P. Zotto, G. Zumerle, A. Braghieri, A. Magnani, P. Montagna, S. P. Ratti, V. Re, M. Ressegotti, C. Riccardi, P. Salvini, I. Vai, P. Vitulo, M. Biasini, G. M. Bilei, C. Cecchi, D. Ciangottini, L. Fanò, P. Lariccia, R. Leonardi, E. Manoni, G. Mantovani, V. Mariani, M. Menichelli, A. Rossi, A. Santocchia, D. Spiga, K. Androsov, P. Azzurri, G. Bagliesi, L. Bianchini, T. Boccali, L. Borrello, R. Castaldi, M. A. Ciocci, R. Dell’Orso, G. Fedi, F. Fiori, L. Giannini, A. Giassi, M. T. Grippo, F. Ligabue, E. Manca, G. Mandorli, A. Messineo, F. Palla, A. Rizzi, P. Spagnolo, R. Tenchini, G. Tonelli, A. Venturi, P. G. Verdini, L. Barone, F. Cavallari, M. Cipriani, D. Del Re, E. Di Marco, M. Diemoz, S. Gelli, E. Longo, B. Marzocchi, P. Meridiani, G. Organtini, F. Pandolfi, R. Paramatti, F. Preiato, S. Rahatlou, C. Rovelli, F. Santanastasio, N. Amapane, R. Arcidiacono, S. Argiro, M. Arneodo, N. Bartosik, R. Bellan, C. Biino, N. Cartiglia, F. Cenna, S. Cometti, M. Costa, R. Covarelli, N. Demaria, B. Kiani, C. Mariotti, S. Maselli, E. Migliore, V. Monaco, E. Monteil, M. Monteno, M. M. Obertino, L. Pacher, N. Pastrone, M. Pelliccioni, G. L. Pinna Angioni, A. Romero, M. Ruspa, R. Sacchi, K. Shchelina, V. Sola, A. Solano, D. Soldi, A. Staiano, S. Belforte, V. Candelise, M. Casarsa, F. Cossutti, A. Da Rold, G. Della Ricca, F. Vazzoler, A. Zanetti, D. H. Kim, G. N. Kim, M. S. Kim, J. Lee, S. Lee, S. W. Lee, C. S. Moon, Y. D. Oh, S. I. Pak, S. Sekmen, D. C. Son, Y. C. Yang, H. Kim, D. H. Moon, G. Oh, B. Francois, J. Goh, T. J. Kim, S. Cho, S. Choi, Y. Go, D. Gyun, S. Ha, B. Hong, Y. Jo, K. Lee, K. S. Lee, S. Lee, J. Lim, S. K. Park, Y. Roh, H. S. Kim, J. Almond, J. Kim, J. S. Kim, H. Lee, K. Lee, K. Nam, S. B. Oh, B. C. Radburn-Smith, S. h. Seo, U. K. Yang, H. D. Yoo, G. B. Yu, D. Jeon, H. Kim, J. H. Kim, J. S. H. Lee, I. C. Park, Y. Choi, C. Hwang, J. Lee, I. Yu, V. Dudenas, A. Juodagalvis, J. Vaitkus, I. Ahmed, Z. A. Ibrahim, M. A. B. Md Ali, F. Mohamad Idris, W. A. T. Wan Abdullah, M. N. Yusli, Z. Zolkapli, J. F. Benitez, A. Castaneda Hernandez, J. A. Murillo Quijada, H. Castilla-Valdez, E. De La Cruz-Burelo, M. C. Duran-Osuna, I. Heredia-De La Cruz, R. I. Rabadan-Trejo, R. Lopez-Fernandez, J. Mejia Guisao, R. I. Rabadan-Trejo, M. Ramirez-Garcia, G. Ramirez-Sanchez, R. Reyes-Almanza, A. Sanchez-Hernandez, S. Carrillo Moreno, C. Oropeza Barrera, F. Vazquez Valencia, J. Eysermans, I. Pedraza, H. A. Salazar Ibarguen, C. Uribe Estrada, A. Morelos Pineda, D. Krofcheck, S. Bheesette, P. H. Butler, A. Ahmad, M. Ahmad, M. I. Asghar, Q. Hassan, H. R. Hoorani, A. Saddique, M. A. Shah, M. Shoaib, M. Waqas, H. Bialkowska, M. Bluj, B. Boimska, T. Frueboes, M. Górski, M. Kazana, M. Szleper, P. Traczyk, P. Zalewski, K. Bunkowski, A. Byszuk, K. Doroba, A. Kalinowski, M. Konecki, J. Krolikowski, M. Misiura, M. Olszewski, A. Pyskir, M. Walczak, M. Araujo, P. Bargassa, C. Beirão Da Cruz E Silva, A. Di Francesco, P. Faccioli, B. Galinhas, M. Gallinaro, J. Hollar, N. Leonardo, M. V. Nemallapudi, J. Seixas, G. Strong, O. Toldaiev, D. Vadruccio, J. Varela, S. Afanasiev, P. Bunin, M. Gavrilenko, I. Golutvin, I. Gorbunov, A. Kamenev, V. Karjavine, A. Lanev, A. Malakhov, V. Matveev, P. Moisenz, V. Palichik, V. Perelygin, S. Shmatov, S. Shulha, N. Skatchkov, V. Smirnov, N. Voytishin, A. Zarubin, V. Golovtsov, Y. Ivanov, V. Kim, E. Kuznetsova, P. Levchenko, V. Murzin, V. Oreshkin, I. Smirnov, D. Sosnov, V. Sulimov, L. Uvarov, S. Vavilov, A. Vorobyev, Yu. Andreev, A. Dermenev, S. Gninenko, N. Golubev, A. Karneyeu, M. Kirsanov, N. Krasnikov, A. Pashenkov, D. Tlisov, A. Toropin, V. Epshteyn, V. Gavrilov, N. Lychkovskaya, V. Popov, I. Pozdnyakov, G. Safronov, A. Spiridonov, A. Stepennov, V. Stolin, M. Toms, E. Vlasov, A. Zhokin, T. Aushev, R. Chistov, M. Danilov, P. Parygin, D. Philippov, S. Polikarpov, E. Tarkovskii, V. Andreev, M. Azarkin, I. Dremin, M. Kirakosyan, S. V. Rusakov, A. Terkulov, A. Baskakov, A. Belyaev, E. Boos, M. Dubinin, L. Dudko, A. Ershov, A. Gribushin, V. Klyukhin, O. Kodolova, I. Lokhtin, I. Miagkov, S. Obraztsov, S. Petrushanko, V. Savrin, A. Snigirev, A. Barnyakov, V. Blinov, T. Dimova, L. Kardapoltsev, Y. Skovpen, I. Azhgirey, I. Bayshev, S. Bitioukov, D. Elumakhov, A. Godizov, V. Kachanov, A. Kalinin, D. Konstantinov, P. Mandrik, V. Petrov, R. Ryutin, S. Slabospitskii, A. Sobol, S. Troshin, N. Tyurin, A. Uzunian, A. Volkov, A. Babaev, S. Baidali, V. Okhotnikov, P. Adzic, P. Cirkovic, D. Devetak, M. Dordevic, J. Milosevic, J. Alcaraz Maestre, A. Álvarez Fernández, I. Bachiller, M. Barrio Luna, J. A. Brochero Cifuentes, M. Cerrada, N. Colino, B. De La Cruz, A. Delgado Peris, C. Fernandez Bedoya, J. P. Fernández Ramos, J. Flix, M. C. Fouz, O. Gonzalez Lopez, S. Goy Lopez, J. M. Hernandez, M. I. Josa, D. Moran, A. Pérez-Calero Yzquierdo, J. Puerta Pelayo, I. Redondo, L. Romero, M. S. Soares, A. Triossi, C. Albajar, J. F. de Trocóniz, J. Cuevas, C. Erice, J. Fernandez Menendez, S. Folgueras, I. Gonzalez Caballero, J. R. González Fernández, E. Palencia Cortezon, V. Rodríguez Bouza, S. Sanchez Cruz, P. Vischia, J. M. Vizan Garcia, I. J. Cabrillo, A. Calderon, B. Chazin Quero, J. Duarte Campderros, M. Fernandez, P. J. Fernández Manteca, A. García Alonso, J. Garcia-Ferrero, G. Gomez, A. Lopez Virto, J. Marco, C. Martinez Rivero, P. Martinez Ruiz del Arbol, F. Matorras, J. Piedra Gomez, C. Prieels, T. Rodrigo, A. Ruiz-Jimeno, L. Scodellaro, N. Trevisani, I. Vila, R. Vilar Cortabitarte, N. Wickramage, D. Abbaneo, B. Akgun, E. Auffray, G. Auzinger, P. Baillon, A. H. Ball, D. Barney, J. Bendavid, M. Bianco, A. Bocci, C. Botta, E. Brondolin, T. Camporesi, M. Cepeda, G. Cerminara, E. Chapon, Y. Chen, G. Cucciati, D. d’Enterria, A. Dabrowski, N. Daci, V. Daponte, A. David, A. De Roeck, N. Deelen, M. Dobson, M. Dünser, N. Dupont, A. Elliott-Peisert, P. Everaerts, F. Fallavollita, D. Fasanella, G. Franzoni, J. Fulcher, W. Funk, D. Gigi, A. Gilbert, K. Gill, F. Glege, M. Guilbaud, D. Gulhan, J. Hegeman, C. Heidegger, V. Innocente, A. Jafari, P. Janot, O. Karacheban, J. Kieseler, A. Kornmayer, M. Krammer, C. Lange, P. Lecoq, C. Lourenço, L. Malgeri, M. Mannelli, F. Meijers, J. A. Merlin, S. Mersi, E. Meschi, P. Milenovic, F. Moortgat, M. Mulders, J. Ngadiuba, S. Nourbakhsh, S. Orfanelli, L. Orsini, F. Pantaleo, L. Pape, E. Perez, M. Peruzzi, A. Petrilli, G. Petrucciani, A. Pfeiffer, M. Pierini, F. M. Pitters, D. Rabady, A. Racz, T. Reis, G. Rolandi, M. Rovere, H. Sakulin, C. Schäfer, C. Schwick, M. Seidel, M. Selvaggi, A. Sharma, P. Silva, P. Sphicas, A. Stakia, J. Steggemann, M. Tosi, D. Treille, A. Tsirou, V. Veckalns, M. Verzetti, W. D. Zeuner, L. Caminada, K. Deiters, W. Erdmann, R. Horisberger, Q. Ingram, H. C. Kaestli, D. Kotlinski, U. Langenegger, T. Rohe, S. A. Wiederkehr, M. Backhaus, L. Bäni, P. Berger, N. Chernyavskaya, G. Dissertori, M. Dittmar, M. Donegà, C. Dorfer, T. A. Gómez Espinosa, C. Grab, D. Hits, T. Klijnsma, W. Lustermann, R. A. Manzoni, M. Marionneau, M. T. Meinhard, F. Micheli, P. Musella, F. Nessi-Tedaldi, J. Pata, F. Pauss, G. Perrin, L. Perrozzi, S. Pigazzini, M. Quittnat, C. Reissel, D. Ruini, D. A. Sanz Becerra, M. Schönenberger, L. Shchutska, V. R. Tavolaro, K. Theofilatos, M. L. Vesterbacka Olsson, R. Wallny, D. H. Zhu, T. K. Aarrestad, C. Amsler, D. Brzhechko, M. F. Canelli, A. De Cosa, R. Del Burgo, S. Donato, C. Galloni, T. Hreus, B. Kilminster, S. Leontsinis, I. Neutelings, G. Rauco, P. Robmann, D. Salerno, K. Schweiger, C. Seitz, Y. Takahashi, A. Zucchetta, Y. H. Chang, K. y. Cheng, T. H. Doan, R. Khurana, C. M. Kuo, W. Lin, A. Pozdnyakov, S. S. Yu, P. Chang, Y. Chao, K. F. Chen, P. H. Chen, W.-S. Hou, Arun Kumar, Y. F. Liu, R.-S. Lu, E. Paganis, A. Psallidas, A. Steen, B. Asavapibhop, N. Srimanobhas, N. Suwonjandee, A. Bat, F. Boran, S. Cerci, S. Damarseckin, Z. S. Demiroglu, F. Dolek, C. Dozen, I. Dumanoglu, S. Girgis, G. Gokbulut, Y. Guler, E. Gurpinar, I. Hos, C. Isik, E. E. Kangal, O. Kara, A. Kayis Topaksu, U. Kiminsu, M. Oglakci, G. Onengut, K. Ozdemir, A. Polatoz, D. Sunar Cerci, B. Tali, U. G. Tok, S. Turkcapar, I. S. Zorbakir, C. Zorbilmez, B. Isildak, G. Karapinar, M. Yalvac, M. Zeyrek, I. O. Atakisi, E. Gülmez, M. Kaya, O. Kaya, S. Ozkorucuklu, S. Tekten, E. A. Yetkin, M. N. Agaras, A. Cakir, K. Cankocak, Y. Komurcu, S. Sen, B. Grynyov, L. Levchuk, F. Ball, L. Beck, J. J. Brooke, D. Burns, E. Clement, D. Cussans, O. Davignon, H. Flacher, J. Goldstein, G. P. Heath, H. F. Heath, L. Kreczko, D. M. Newbold, S. Paramesvaran, B. Penning, T. Sakuma, D. Smith, V. J. Smith, J. Taylor, A. Titterton, K. W. Bell, A. Belyaev, C. Brew, R. M. Brown, D. Cieri, D. J. A. Cockerill, J. A. Coughlan, K. Harder, S. Harper, J. Linacre, E. Olaiya, D. Petyt, C. H. Shepherd-Themistocleous, A. Thea, I. R. Tomalin, T. Williams, W. J. Womersley, R. Bainbridge, P. Bloch, J. Borg, S. Breeze, O. Buchmuller, A. Bundock, D. Colling, P. Dauncey, G. Davies, M. Della Negra, R. Di Maria, G. Hall, G. Iles, T. James, M. Komm, C. Laner, L. Lyons, A.-M. Magnan, S. Malik, A. Martelli, J. Nash, A. Nikitenko, V. Palladino, M. Pesaresi, D. M. Raymond, A. Richards, A. Rose, E. Scott, C. Seez, A. Shtipliyski, G. Singh, M. Stoye, T. Strebler, S. Summers, A. Tapper, K. Uchida, T. Virdee, N. Wardle, D. Winterbottom, J. Wright, S. C. Zenz, J. E. Cole, P. R. Hobson, A. Khan, P. Kyberd, C. K. Mackay, A. Morton, I. D. Reid, L. Teodorescu, S. Zahid, K. Call, J. Dittmann, K. Hatakeyama, H. Liu, C. Madrid, B. Mcmaster, N. Pastika, C. Smith, R. Bartek, A. Dominguez, A. Buccilli, S. I. Cooper, C. Henderson, P. Rumerio, C. West, D. Arcaro, T. Bose, D. Gastler, D. Pinna, D. Rankin, C. Richardson, J. Rohlf, L. Sulak, D. Zou, G. Benelli, X. Coubez, D. Cutts, M. Hadley, J. Hakala, U. Heintz, J. M. Hogan, K. H. M. Kwok, E. Laird, G. Landsberg, J. Lee, Z. Mao, M. Narain, S. Sagir, R. Syarif, E. Usai, D. Yu, R. Band, C. Brainerd, R. Breedon, D. Burns, M. Calderon De La Barca Sanchez, M. Chertok, J. Conway, R. Conway, P. T. Cox, R. Erbacher, C. Flores, G. Funk, W. Ko, O. Kukral, R. Lander, M. Mulhearn, D. Pellett, J. Pilot, S. Shalhout, M. Shi, D. Stolp, D. Taylor, K. Tos, M. Tripathi, Z. Wang, F. Zhang, M. Bachtis, C. Bravo, R. Cousins, A. Dasgupta, A. Florent, J. Hauser, M. Ignatenko, N. Mccoll, S. Regnard, D. Saltzberg, C. Schnaible, V. Valuev, E. Bouvier, K. Burt, R. Clare, J. W. Gary, S. M. A. Ghiasi Shirazi, G. Hanson, G. Karapostoli, E. Kennedy, F. Lacroix, O. R. Long, M. Olmedo Negrete, M. I. Paneva, W. Si, L. Wang, H. Wei, S. Wimpenny, B. R. Yates, J. G. Branson, P. Chang, S. Cittolin, M. Derdzinski, R. Gerosa, D. Gilbert, B. Hashemi, A. Holzner, D. Klein, G. Kole, V. Krutelyov, J. Letts, M. Masciovecchio, D. Olivito, S. Padhi, M. Pieri, M. Sani, V. Sharma, S. Simon, M. Tadel, A. Vartak, S. Wasserbaech, J. Wood, F. Würthwein, A. Yagil, G. Zevi Della Porta, N. Amin, R. Bhandari, J. Bradmiller-Feld, C. Campagnari, M. Citron, A. Dishaw, V. Dutta, M. Franco Sevilla, L. Gouskos, R. Heller, J. Incandela, A. Ovcharova, H. Qu, J. Richman, D. Stuart, I. Suarez, S. Wang, J. Yoo, D. Anderson, A. Bornheim, J. M. Lawhorn, H. B. Newman, T. Q. Nguyen, M. Spiropulu, J. R. Vlimant, R. Wilkinson, S. Xie, Z. Zhang, R. Y. Zhu, M. B. Andrews, T. Ferguson, T. Mudholkar, M. Paulini, M. Sun, I. Vorobiev, M. Weinberg, J. P. Cumalat, W. T. Ford, F. Jensen, A. Johnson, M. Krohn, E. MacDonald, T. Mulholland, R. Patel, A. Perloff, K. Stenson, K. A. Ulmer, S. R. Wagner, J. Alexander, J. Chaves, Y. Cheng, J. Chu, A. Datta, K. Mcdermott, N. Mirman, J. R. Patterson, D. Quach, A. Rinkevicius, A. Ryd, L. Skinnari, L. Soffi, S. M. Tan, Z. Tao, J. Thom, J. Tucker, P. Wittich, M. Zientek, S. Abdullin, M. Albrow, M. Alyari, G. Apollinari, A. Apresyan, A. Apyan, S. Banerjee, L. A. T. Bauerdick, A. Beretvas, J. Berryhill, P. C. Bhat, K. Burkett, J. N. Butler, A. Canepa, G. B. Cerati, H. W. K. Cheung, F. Chlebana, M. Cremonesi, J. Duarte, V. D. Elvira, J. Freeman, Z. Gecse, E. Gottschalk, L. Gray, D. Green, S. Grünendahl, O. Gutsche, J. Hanlon, R. M. Harris, S. Hasegawa, J. Hirschauer, Z. Hu, B. Jayatilaka, S. Jindariani, M. Johnson, U. Joshi, B. Klima, M. J. Kortelainen, B. Kreis, S. Lammel, D. Lincoln, R. Lipton, M. Liu, T. Liu, J. Lykken, K. Maeshima, J. M. Marraffino, D. Mason, P. McBride, P. Merkel, S. Mrenna, S. Nahn, V. O’Dell, K. Pedro, C. Pena, O. Prokofyev, G. Rakness, L. Ristori, A. Savoy-Navarro, B. Schneider, E. Sexton-Kennedy, A. Soha, W. J. Spalding, L. Spiegel, S. Stoynev, J. Strait, N. Strobbe, L. Taylor, S. Tkaczyk, N. V. Tran, L. Uplegger, E. W. Vaandering, C. Vernieri, M. Verzocchi, R. Vidal, M. Wang, H. A. Weber, A. Whitbeck, D. Acosta, P. Avery, P. Bortignon, D. Bourilkov, A. Brinkerhoff, L. Cadamuro, A. Carnes, M. Carver, D. Curry, R. D. Field, S. V. Gleyzer, B. M. Joshi, J. Konigsberg, A. Korytov, K. H. Lo, P. Ma, K. Matchev, H. Mei, G. Mitselmakher, D. Rosenzweig, K. Shi, D. Sperka, J. Wang, S. Wang, X. Zuo, Y. R. Joshi, S. Linn, A. Ackert, T. Adams, A. Askew, S. Hagopian, V. Hagopian, K. F. Johnson, T. Kolberg, G. Martinez, T. Perry, H. Prosper, A. Saha, C. Schiber, R. Yohay, M. M. Baarmand, V. Bhopatkar, S. Colafranceschi, M. Hohlmann, D. Noonan, M. Rahmani, T. Roy, F. Yumiceva, M. R. Adams, L. Apanasevich, D. Berry, R. R. Betts, R. Cavanaugh, X. Chen, S. Dittmer, O. Evdokimov, C. E. Gerber, D. A. Hangal, D. J. Hofman, K. Jung, J. Kamin, C. Mills, I. D. Sandoval Gonzalez, M. B. Tonjes, H. Trauger, N. Varelas, H. Wang, X. Wang, Z. Wu, J. Zhang, M. Alhusseini, B. Bilki, W. Clarida, K. Dilsiz, S. Durgut, R. P. Gandrajula, M. Haytmyradov, V. Khristenko, J.-P. Merlo, A. Mestvirishvili, A. Moeller, J. Nachtman, H. Ogul, Y. Onel, F. Ozok, A. Penzo, C. Snyder, E. Tiras, J. Wetzel, B. Blumenfeld, A. Cocoros, N. Eminizer, D. Fehling, L. Feng, A. V. Gritsan, W. T. Hung, P. Maksimovic, J. Roskes, U. Sarica, M. Swartz, M. Xiao, C. You, A. Al-bataineh, P. Baringer, A. Bean, S. Boren, J. Bowen, A. Bylinkin, J. Castle, S. Khalil, A. Kropivnitskaya, D. Majumder, W. Mcbrayer, M. Murray, C. Rogan, S. Sanders, E. Schmitz, J. D. Tapia Takaki, Q. Wang, S. Duric, A. Ivanov, K. Kaadze, D. Kim, Y. Maravin, D. R. Mendis, T. Mitchell, A. Modak, A. Mohammadi, L. K. Saini, N. Skhirtladze, F. Rebassoo, D. Wright, A. Baden, O. Baron, A. Belloni, S. C. Eno, Y. Feng, C. Ferraioli, N. J. Hadley, S. Jabeen, G. Y. Jeng, R. G. Kellogg, J. Kunkle, A. C. Mignerey, S. Nabili, F. Ricci-Tam, Y. H. Shin, A. Skuja, S. C. Tonwar, K. Wong, D. Abercrombie, B. Allen, V. Azzolini, A. Baty, G. Bauer, R. Bi, S. Brandt, W. Busza, I. A. Cali, M. D’Alfonso, Z. Demiragli, G. Gomez Ceballos, M. Goncharov, P. Harris, D. Hsu, M. Hu, Y. Iiyama, G. M. Innocenti, M. Klute, D. Kovalskyi, Y.-J. Lee, P. D. Luckey, B. Maier, A. C. Marini, C. Mcginn, C. Mironov, S. Narayanan, X. Niu, C. Paus, C. Roland, G. Roland, G. S. F. Stephans, K. Sumorok, K. Tatar, D. Velicanu, J. Wang, T. W. Wang, B. Wyslouch, S. Zhaozhong, A. C. Benvenuti, R. M. Chatterjee, A. Evans, P. Hansen, J. Hiltbrand, Sh. Jain, S. Kalafut, Y. Kubota, Z. Lesko, J. Mans, N. Ruckstuhl, R. Rusack, M. A. Wadud, J. G. Acosta, S. Oliveros, E. Avdeeva, K. Bloom, D. R. Claes, C. Fangmeier, F. Golf, R. Gonzalez Suarez, R. Kamalieddin, I. Kravchenko, J. Monroy, J. E. Siado, G. R. Snow, B. Stieger, A. Godshalk, C. Harrington, I. Iashvili, A. Kharchilava, C. Mclean, D. Nguyen, A. Parker, S. Rappoccio, B. Roozbahani, G. Alverson, E. Barberis, C. Freer, Y. Haddad, A. Hortiangtham, D. M. Morse, T. Orimoto, R. Teixeira De Lima, T. Wamorkar, B. Wang, A. Wisecarver, D. Wood, S. Bhattacharya, O. Charaf, K. A. Hahn, N. Mucia, N. Odell, M. H. Schmitt, K. Sung, M. Trovato, M. Velasco, R. Bucci, N. Dev, M. Hildreth, K. Hurtado Anampa, C. Jessop, D. J. Karmgard, N. Kellams, K. Lannon, W. Li, N. Loukas, N. Marinelli, F. Meng, C. Mueller, Y. Musienko, M. Planer, A. Reinsvold, R. Ruchti, P. Siddireddy, G. Smith, S. Taroni, M. Wayne, A. Wightman, M. Wolf, A. Woodard, J. Alimena, L. Antonelli, B. Bylsma, L. S. Durkin, S. Flowers, B. Francis, A. Hart, C. Hill, W. Ji, T. Y. Ling, W. Luo, B. L. Winer, S. Cooperstein, P. Elmer, J. Hardenbrook, S. Higginbotham, A. Kalogeropoulos, D. Lange, M. T. Lucchini, J. Luo, D. Marlow, K. Mei, I. Ojalvo, J. Olsen, C. Palmer, P. Piroué, J. Salfeld-Nebgen, D. Stickland, C. Tully, S. Malik, S. Norberg, A. Barker, V. E. Barnes, S. Das, L. Gutay, M. Jones, A. W. Jung, A. Khatiwada, B. Mahakud, D. H. Miller, N. Neumeister, C. C. Peng, S. Piperov, H. Qiu, J. F. Schulte, J. Sun, F. Wang, R. Xiao, W. Xie, T. Cheng, J. Dolen, N. Parashar, Z. Chen, K. M. Ecklund, S. Freed, F. J. M. Geurts, M. Kilpatrick, W. Li, B. P. Padley, R. Redjimi, J. Roberts, J. Rorie, W. Shi, Z. Tu, J. Zabel, A. Zhang, A. Bodek, P. de Barbaro, R. Demina, Y. t. Duh, J. L. Dulemba, C. Fallon, T. Ferbel, M. Galanti, A. Garcia-Bellido, J. Han, O. Hindrichs, A. Khukhunaishvili, P. Tan, R. Taus, A. Agapitos, J. P. Chou, Y. Gershtein, E. Halkiadakis, M. Heindl, E. Hughes, S. Kaplan, R. Kunnawalkam Elayavalli, S. Kyriacou, A. Lath, R. Montalvo, K. Nash, M. Osherson, H. Saka, S. Salur, S. Schnetzer, D. Sheffield, S. Somalwar, R. Stone, S. Thomas, P. Thomassen, M. Walker, A. G. Delannoy, J. Heideman, G. Riley, S. Spanier, O. Bouhali, A. Celik, M. Dalchenko, M. De Mattia, A. Delgado, S. Dildick, R. Eusebi, J. Gilmore, T. Huang, T. Kamon, S. Luo, R. Mueller, D. Overton, L. Perniè, D. Rathjens, A. Safonov, N. Akchurin, J. Damgov, F. De Guio, P. R. Dudero, S. Kunori, K. Lamichhane, S. W. Lee, T. Mengke, S. Muthumuni, T. Peltola, S. Undleeb, I. Volobouev, Z. Wang, S. Greene, A. Gurrola, R. Janjam, W. Johns, C. Maguire, A. Melo, H. Ni, K. Padeken, J. D. Ruiz Alvarez, P. Sheldon, S. Tuo, J. Velkovska, M. Verweij, Q. Xu, M. W. Arenton, P. Barria, B. Cox, R. Hirosky, M. Joyce, A. Ledovskoy, H. Li, C. Neu, T. Sinthuprasith, Y. Wang, E. Wolfe, F. Xia, R. Harr, P. E. Karchin, N. Poudyal, J. Sturdy, P. Thapa, S. Zaleski, M. Brodski, J. Buchanan, C. Caillol, D. Carlsmith, S. Dasu, L. Dodd, B. Gomber, M. Grothe, M. Herndon, A. Hervé, U. Hussain, P. Klabbers, A. Lanaro, K. Long, R. Loveless, T. Ruggles, A. Savin, V. Sharma, N. Smith, W. H. Smith, N. Woods, CMS Collaboration

**Affiliations:** 1 Yerevan Physics Institute, Yerevan, Armenia; 2 Institut für Hochenergiephysik, Wien, Austria; 3 Institute for Nuclear Problems, Minsk, Belarus; 4 Universiteit Antwerpen, Antwerpen, Belgium; 5 Vrije Universiteit Brussel, Brussel, Belgium; 6 Université Libre de Bruxelles, Brussels, Belgium; 7 Ghent University, Ghent, Belgium; 8 Université Catholique de Louvain, Louvain-la-Neuve, Belgium; 9 Centro Brasileiro de Pesquisas Fisicas, Rio de Janeiro, Brazil; 10 Universidade do Estado do Rio de Janeiro, Rio de Janeiro, Brazil; 11 Universidade Estadual Paulista, Universidade Federal do ABC, São Paulo, Brazil; 12 Institute for Nuclear Research and Nuclear Energy, Bulgarian Academy of Sciences, Sofia, Bulgaria; 13 University of Sofia, Sofia, Bulgaria; 14 Beihang University, Beijing, China; 15 Institute of High Energy Physics, Beijing, China; 16 State Key Laboratory of Nuclear Physics and Technology, Peking University, Beijing, China; 17 Tsinghua University, Beijing, China; 18 Universidad de Los Andes, Bogota, Colombia; 19 University of Split, Faculty of Electrical Engineering, Mechanical Engineering and Naval Architecture, Split, Croatia; 20 University of Split, Faculty of Science, Split, Croatia; 21 Institute Rudjer Boskovic, Zagreb, Croatia; 22 University of Cyprus, Nicosia, Cyprus; 23 Charles University, Prague, Czech Republic; 24 Escuela Politecnica Nacional, Quito, Ecuador; 25 Universidad San Francisco de Quito, Quito, Ecuador; 26 Academy of Scientific Research and Technology of the Arab Republic of Egypt, Egyptian Network of High Energy Physics, Cairo, Egypt; 27 National Institute of Chemical Physics and Biophysics, Tallinn, Estonia; 28 Department of Physics, University of Helsinki, Helsinki, Finland; 29 Helsinki Institute of Physics, Helsinki, Finland; 30 Lappeenranta University of Technology, Lappeenranta, Finland; 31 IRFU, CEA, Université Paris-Saclay, Gif-sur-Yvette, France; 32 Laboratoire Leprince-Ringuet, Ecole polytechnique, CNRS/IN2P3, Université Paris-Saclay, Palaiseau, France; 33 Université de Strasbourg, CNRS, IPHC UMR 7178, Strasbourg, France; 34 Centre de Calcul de l’Institut National de Physique Nucleaire et de Physique des Particules, CNRS/IN2P3, Villeurbanne, France; 35 Université de Lyon, Université Claude Bernard Lyon 1, CNRS-IN2P3, Institut de Physique Nucléaire de Lyon, Villeurbanne, France; 36 Georgian Technical University, Tbilisi, Georgia; 37 Tbilisi State University, Tbilisi, Georgia; 38 RWTH Aachen University, I. Physikalisches Institut, Aachen, Germany; 39 RWTH Aachen University, III. Physikalisches Institut A, Aachen, Germany; 40 RWTH Aachen University, III. Physikalisches Institut B, Aachen, Germany; 41 Deutsches Elektronen-Synchrotron, Hamburg, Germany; 42 University of Hamburg, Hamburg, Germany; 43 Karlsruher Institut fuer Technologie, Karlsruhe, Germany; 44 Institute of Nuclear and Particle Physics (INPP), NCSR Demokritos, Agia Paraskevi, Greece; 45 National and Kapodistrian University of Athens, Athens, Greece; 46 National Technical University of Athens, Athens, Greece; 47 University of Ioánnina, Ioannina, Greece; 48 MTA-ELTE Lendület CMS Particle and Nuclear Physics Group, Eötvös Loránd University, Budapest, Hungary; 49 Wigner Research Centre for Physics, Budapest, Hungary; 50 Institute of Nuclear Research ATOMKI, Debrecen, Hungary; 51 Institute of Physics, University of Debrecen, Debrecen, Hungary; 52 Indian Institute of Science (IISc), Bangalore, India; 53 National Institute of Science Education and Research, HBNI, Bhubaneswar, India; 54 Panjab University, Chandigarh, India; 55 University of Delhi, Delhi, India; 56 Saha Institute of Nuclear Physics, HBNI, Kolkata, India; 57 Indian Institute of Technology Madras, Madras, India; 58 Bhabha Atomic Research Centre, Mumbai, India; 59 Tata Institute of Fundamental Research-A, Mumbai, India; 60 Tata Institute of Fundamental Research-B, Mumbai, India; 61 Indian Institute of Science Education and Research (IISER), Pune, India; 62 Institute for Research in Fundamental Sciences (IPM), Tehran, Iran; 63 University College Dublin, Dublin, Ireland; 64 INFN Sezione di Bari, Università di Bari, Politecnico di Bari, Bari, Italy; 65 INFN Sezione di Bologna, Università di Bologna, Bologna, Italy; 66 INFN Sezione di Catania, Università di Catania, Catania, Italy; 67 INFN Sezione di Firenze, Università di Firenze, Firenze, Italy; 68 INFN Laboratori Nazionali di Frascati, Frascati, Italy; 69 INFN Sezione di Genova, Università di Genova, Genova, Italy; 70 INFN Sezione di Milano-Bicocca, Università di Milano-Bicocca, Milan, Italy; 71 INFN Sezione di Napoli, Università di Napoli ’Federico II’ , Napoli, Italy, Università della Basilicata, Potenza, Italy, Università G. Marconi, Rome, Italy; 72 INFN Sezione di Padova, Università di Padova, Padova, Italy, Università di Trento, Trento, Italy; 73 INFN Sezione di Pavia, Università di Pavia, Pavia, Italy; 74 INFN Sezione di Perugia, Università di Perugia, Perugia, Italy; 75 INFN Sezione di Pisa, Università di Pisa, Scuola Normale Superiore di Pisa, Pisa, Italy; 76 INFN Sezione di Roma, Sapienza Università di Roma, Rome, Italy; 77 INFN Sezione di Torino, Università di Torino, Torino, Italy, Università del Piemonte Orientale, Novara, Italy; 78 INFN Sezione di Trieste, Università di Trieste, Trieste, Italy; 79 Kyungpook National University, Daegu, South Korea; 80 Chonnam National University, Institute for Universe and Elementary Particles, Kwangju, South Korea; 81 Hanyang University, Seoul, South Korea; 82 Korea University, Seoul, South Korea; 83 Sejong University, Seoul, South Korea; 84 Seoul National University, South Seoul, Korea; 85 University of Seoul, South Seoul, Korea; 86 Sungkyunkwan University, Suwon, South Korea; 87 Vilnius University, Vilnius, Lithuania; 88 National Centre for Particle Physics, Universiti Malaya, Kuala Lumpur, Malaysia; 89 Universidad de Sonora (UNISON), Hermosillo, Mexico; 90 Centro de Investigacion y de Estudios Avanzados del IPN, Mexico City, Mexico; 91 Universidad Iberoamericana, Mexico City, Mexico; 92 Benemerita Universidad Autonoma de Puebla, Puebla, Mexico; 93 Universidad Autónoma de San Luis Potosí, San Luis Potosí, Mexico; 94 University of Auckland, Auckland, New Zealand; 95 University of Canterbury, Christchurch, New Zealand; 96 National Centre for Physics, Quaid-I-Azam University, Islamabad, Pakistan; 97 National Centre for Nuclear Research, Swierk, Poland; 98 Institute of Experimental Physics, Faculty of Physics, University of Warsaw, Warsaw, Poland; 99 Laboratório de Instrumentação e Física Experimental de Partículas, Lisbon, Portugal; 100 Joint Institute for Nuclear Research, Dubna, Russia; 101 Petersburg Nuclear Physics Institute, Gatchina (St. Petersburg), Russia; 102 Institute for Nuclear Research, Moscow, Russia; 103 Institute for Theoretical and Experimental Physics, Moscow, Russia; 104 Moscow Institute of Physics and Technology, Moscow, Russia; 105 National Research Nuclear University ’Moscow Engineering Physics Institute’ (MEPhI), Moscow, Russia; 106 P.N. Lebedev Physical Institute, Moscow, Russia; 107 Skobeltsyn Institute of Nuclear Physics, Lomonosov Moscow State University, Moscow, Russia; 108 Novosibirsk State University (NSU), Novosibirsk, Russia; 109 Institute for High Energy Physics of National Research Centre ’Kurchatov Institute’, Protvino, Russia; 110 National Research Tomsk Polytechnic University, Tomsk, Russia; 111 University of Belgrade, Faculty of Physics and Vinca Institute of Nuclear Sciences, Belgrade, Serbia; 112 Centro de Investigaciones Energéticas Medioambientales y Tecnológicas (CIEMAT), Madrid, Spain; 113 Universidad Autónoma de Madrid, Madrid, Spain; 114 Universidad de Oviedo, Oviedo, Spain; 115 Instituto de Física de Cantabria (IFCA), CSIC-Universidad de Cantabria, Santander, Spain; 116 University of Ruhuna, Department of Physics, Matara, Sri Lanka; 117 CERN, European Organization for Nuclear Research, Geneva, Switzerland; 118 Paul Scherrer Institut, Villigen, Switzerland; 119 ETH Zurich, Institute for Particle Physics and Astrophysics (IPA), Zurich, Switzerland; 120 Universität Zürich, Zurich, Switzerland; 121 National Central University, Chung-Li, Taiwan; 122 National Taiwan University (NTU), Taipei, Taiwan; 123 Chulalongkorn University, Faculty of Science, Department of Physics, Bangkok, Thailand; 124 Çukurova University, Physics Department, Science and Art Faculty, Adana, Turkey; 125 Middle East Technical University, Physics Department, Ankara, Turkey; 126 Bogazici University, Istanbul, Turkey; 127 Istanbul Technical University, Istanbul, Turkey; 128 Institute for Scintillation Materials of National Academy of Science of Ukraine, Kharkov, Ukraine; 129 National Scientific Center, Kharkov Institute of Physics and Technology, Kharkov, Ukraine; 130 University of Bristol, Bristol, United Kingdom; 131 Rutherford Appleton Laboratory, Didcot, United Kingdom; 132 Imperial College, London, United Kingdom; 133 Brunel University, Uxbridge, United Kingdom; 134 Baylor University, Waco, USA; 135 Catholic University of America, Washington DC, USA; 136 The University of Alabama, Tuscaloosa, USA; 137 Boston University, Boston, USA; 138 Brown University, Providence, USA; 139 University of California, Davis, Davis USA; 140 University of California, Los Angeles, USA; 141 University of California, Riverside, Riverside, USA; 142 University of California, San Diego, La Jolla, USA; 143 University of California, Santa Barbara, Department of Physics, Santa Barbara, USA; 144 California Institute of Technology, Pasadena, USA; 145 Carnegie Mellon University, Pittsburgh, USA; 146 University of Colorado Boulder, Boulder, USA; 147 Cornell University, Ithaca, USA; 148 Fermi National Accelerator Laboratory, Batavia, USA; 149 University of Florida, Gainesville, USA; 150 Florida International University, Miami, USA; 151 Florida State University, Tallahassee, USA; 152 Florida Institute of Technology, Melbourne, USA; 153 University of Illinois at Chicago (UIC), Chicago, USA; 154 The University of Iowa, Iowa City, USA; 155 Johns Hopkins University, Baltimore, USA; 156 The University of Kansas, Lawrence, USA; 157 Kansas State University, Manhattan, USA; 158 Lawrence Livermore National Laboratory, Livermore, USA; 159 University of Maryland, College Park, USA; 160 Massachusetts Institute of Technology, Cambridge, USA; 161 University of Minnesota, Minneapolis, USA; 162 University of Mississippi, Oxford, USA; 163 University of Nebraska-Lincoln, Lincoln, USA; 164 State University of New York at Buffalo, Buffalo, USA; 165 Northeastern University, Boston, USA; 166 Northwestern University, Evanston, USA; 167 University of Notre Dame, Notre Dame, USA; 168 The Ohio State University, Columbus, USA; 169 Princeton University, Princeton, USA; 170 University of Puerto Rico, Mayaguez, USA; 171 Purdue University, West Lafayette, USA; 172 Purdue University Northwest, Hammond, USA; 173 Rice University, Houston, USA; 174 University of Rochester, Rochester, USA; 175 Rutgers, The State University of New Jersey, Piscataway, USA; 176 University of Tennessee, Knoxville, USA; 177 Texas A & M University, College Station, USA; 178 Texas Tech University, Lubbock, USA; 179 Vanderbilt University, Nashville, USA; 180 University of Virginia, Charlottesville, USA; 181 Wayne State University, Detroit, USA; 182 University of Wisconsin, Madison, Madison, WI USA; 183 CERN, 1211 Geneva 23, Switzerland

**Keywords:** CMS, Physics, b hadrons, Heavy flavour spectroscopy, Hadron spectroscopy, Experimental results

## Abstract

Measurements of ${\mathrm {B}} ^{*}_{{\mathrm {s}}2}(5840)^0$ and ${\mathrm {B}} _{{\mathrm {s}}1}(5830)^0$ mesons are performed using a data sample of proton-proton collisions corresponding to an integrated luminosity of , collected with the CMS detector at the LHC at a centre-of-mass energy of $8\,\text {TeV}$. The analysis studies *P*-wave ${\mathrm {B}} ^0_{\mathrm {s}}$ meson decays into ${\mathrm {B}} ^{(*)+} \mathrm {K} ^-$ and ${\mathrm {B}} ^{(*)0} \mathrm {K} ^0_{\mathrm {S}}$, where the ${\mathrm {B}} ^+$ and ${\mathrm {B}} ^0$ mesons are identified using the decays ${\mathrm {B}} ^+ \rightarrow {\mathrm {J}}/\psi \mathrm {K} ^+$ and ${\mathrm {B}} ^0 \rightarrow {\mathrm {J}}/\psi \mathrm {K} ^{*}(892)^0$. The masses of the *P*-wave ${\mathrm {B}} ^0_{\mathrm {s}}$ meson states are measured and the natural width of the ${\mathrm {B}} ^{*}_{{\mathrm {s}}2}(5840)^0$ state is determined. The first measurement of the mass difference between the charged and neutral ${\mathrm {B}} ^{*}$ mesons is also presented. The ${\mathrm {B}} ^{*}_{{\mathrm {s}}2}(5840)^0$ decay to ${\mathrm {B}} ^0 \mathrm {K} ^0_{\mathrm {S}}$ is observed, together with a measurement of its branching fraction relative to the ${\mathrm {B}} ^{*}_{{\mathrm {s}}2}(5840)^0 \rightarrow {\mathrm {B}} ^+ \mathrm {K} ^-$ decay.

## Introduction

The *P*-wave ${\mathrm {B}} ^0_{\mathrm {s}}$ states are the bound states of ${\mathrm {b}}$ and ${\mathrm {s}}$ quarks with an orbital angular momentum $L=1$. Since the b quark is considerably heavier than the strange quark, heavy-quark effective theory (HQET) [1, 2] can be applied to describe this system. In the HQET framework, the state can be described by *L* and the spin of the light quark, providing a total angular momentum of the light subsystem $j=L\pm \frac{1}{2}$. In the case of $L=1$, this results in $j=\frac{1}{2}$ or $j=\frac{3}{2}$. Including the additional splitting from the spin of the heavy b quark results in a total angular momentum $J=j\pm \frac{1}{2}$, yielding two doublets, with the four states denoted as: ${\mathrm {B}} ^{*}_{{\mathrm {s}}0}$ ($j=\frac{1}{2}$, $J^P=0^+$), ${\mathrm {B}} ^{*}_{{\mathrm {s}}1}$ ($j=\frac{1}{2}$, $J^P=1^+$), ${\mathrm {B}} _{{\mathrm {s}}1}$ ($j=\frac{3}{2}$, $J^P=1^+$), and ${\mathrm {B}} ^{*}_{{\mathrm {s}}2}$ ($j=\frac{3}{2}$, $J^P=2^+$). The two former states have not been observed to date, while the latter two are known as the ${\mathrm {B}} _{{\mathrm {s}}1}(5830)^0$ and ${\mathrm {B}} ^{*}_{{\mathrm {s}}2}(5840)^0$ mesons, respectively. For simplicity in this paper, shortened symbols are used to denote the following particles: $\mathrm {K} ^{*0} \equiv \mathrm {K} ^{*}(892)^0$, ${\mathrm {B}} _{1} \equiv {\mathrm {B}} _{1}(5721)^0$, ${\mathrm {B}} ^{*}_{2} \equiv {\mathrm {B}} ^{*}_{2}(5747)^0$, ${\mathrm {B}} _{{\mathrm {s}}1} \equiv {\mathrm {B}} _{{\mathrm {s}}1}(5830)^0$, ${\mathrm {B}} ^{*}_{{\mathrm {s}}2} \equiv {\mathrm {B}} ^{*}_{{\mathrm {s}}2}(5840)^0$, and ${\mathrm {B}} ^{(*)}_{{\mathrm {s}}1,2}$ refers to either ${\mathrm {B}} _{{\mathrm {s}}1}$ or ${\mathrm {B}} ^{*}_{{\mathrm {s}}2}$. Charge-conjugate states are implied throughout the paper. According to HQET, the decays ${\mathrm {B}} ^{*}_{{\mathrm {s}}2} \rightarrow {\mathrm {B}} ^+ \mathrm {K} ^-$, ${\mathrm {B}} ^{*}_{{\mathrm {s}}2} \rightarrow {\mathrm {B}} ^{*+} \mathrm {K} ^-$, and ${\mathrm {B}} _{{\mathrm {s}}1} \rightarrow {\mathrm {B}} ^{*+} \mathrm {K} ^-$ are allowed and should proceed through a *D*-wave transition, while the decay ${\mathrm {B}} _{{\mathrm {s}}1} \rightarrow {\mathrm {B}} ^+ \mathrm {K} ^-$ is forbidden. Similar conclusions apply to the decays into ${\mathrm {B}} ^{(*)0} \mathrm {K} ^0_{\mathrm {S}}$.

Orbitally excited states of the ${\mathrm {B}} ^0_{\mathrm {s}}$ meson were observed by the CDF and D0 Collaborations via the decays into ${\mathrm {B}} ^{(*)+} \mathrm {K} ^-$ [3, 4]. More recently, the LHCb Collaboration presented a more precise study of these states and observed the decay ${\mathrm {B}} ^{*}_{{\mathrm {s}}2}(5840)^0 \rightarrow {\mathrm {B}} ^{*+} \mathrm {K} ^-$ [5], favouring the spin-parity assignment $J^P=2^+$ for the ${\mathrm {B}} ^{*}_{{\mathrm {s}}2}(5840)^0$ state. The CDF Collaboration subsequently presented a study of excited ${\mathrm {B}}$ meson states [6] that included measurements of the ${\mathrm {B}} ^{(*)}_{{\mathrm {s}}1,2} \rightarrow {\mathrm {B}} ^{(*)+} \mathrm {K} ^-$ decays. Table 1 summarizes all the available experimental ${\mathrm {B}} ^{(*)}_{{\mathrm {s}}1,2}$ results.

**Table 1:** Results on the masses, mass differences, and natural widths of the ${\mathrm {B}} ^{(*)}_{{\mathrm {s}}1,2}$ mesons from previous measurements. The mass differences are defined as $\varDelta M_{{\mathrm {B}} _{{\mathrm {s}}1}}^{\pm } \equiv M({\mathrm {B}} _{{\mathrm {s}}1})-M_{{\mathrm {B}} ^{*+}}^{\mathrm {PDG}}-M_{\mathrm {K} ^-}^{\mathrm {PDG}}$ and $\varDelta M_{{\mathrm {B}} ^{*}_{{\mathrm {s}}2}}^{\pm } \equiv M({\mathrm {B}} ^{*}_{{\mathrm {s}}2})-M_{{\mathrm {B}} ^+}^{\mathrm {PDG}}-M_{\mathrm {K} ^-}^{\mathrm {PDG}}$, where the PDG superscript refers to the world-average mass values at the time of each publication

	CDF [3]	D0 [4]	LHCb [5]	CDF [6]
$M({\mathrm {B}} ^{*}_{{\mathrm {s}}2})\,[\,\text {MeV} ]$	$5839.6\pm 0.7$	$5839.6\pm 1.3$	$5839.99\pm 0.21$	$5839.7\pm 0.2$
$M({\mathrm {B}} _{{\mathrm {s}}1})\,[\,\text {MeV} ]$	$5829.4\pm 0.7$	−	$5828.40\pm 0.41$	$5828.3\pm 0.5$
$\varDelta M_{{\mathrm {B}} _{{\mathrm {s}}1}}^{\pm } \,[\,\text {MeV} ]$	$10.73\pm 0.25$	$11.5\pm 1.4$	$10.46\pm 0.06$	$10.35\pm 0.19$
$\varDelta M_{{\mathrm {B}} ^{*}_{{\mathrm {s}}2}}^{\pm } \,[\,\text {MeV} ]$	$66.96\pm 0.41$	$66.7\pm 1.1$	$67.06\pm 0.12$	$66.73\pm 0.19$
$\varGamma ({\mathrm {B}} ^{*}_{{\mathrm {s}}2})\,[\,\text {MeV} ]$	–	–	$1.56\pm 0.49$	$1.4\pm 0.4$
$\varGamma ({\mathrm {B}} _{{\mathrm {s}}1})\,[\,\text {MeV} ]$	–	–	–	$0.5\pm 0.4$

In this paper, the first observation of the ${\mathrm {B}} ^{*}_{{\mathrm {s}}2} \rightarrow {\mathrm {B}} ^0 \mathrm {K} ^0_{\mathrm {S}}$ decay and a measurement of its branching fraction relative to that of the ${\mathrm {B}} ^{*}_{{\mathrm {s}}2} \rightarrow {\mathrm {B}} ^+ \mathrm {K} ^-$ decay are presented. The ${\mathrm {B}} ^+$ and ${\mathrm {B}} ^0$ candidates are reconstructed using the ${\mathrm {B}} ^+ \rightarrow {\mathrm {J}}/\psi (\mu ^+\mu ^-)\mathrm {K} ^+$ and ${\mathrm {B}} ^0 \rightarrow {\mathrm {J}}/\psi (\mu ^+\mu ^-)\mathrm {K} ^{*0} (\mathrm {K} ^+ \pi ^-)$ decays, respectively. Measurements of several ratios of branching fractions and ratios of production cross sections times branching fractions are determined using the formulae:

1 $$\begin{aligned} R^{0\pm }_{2}= & {} \frac{\mathcal {B}({\mathrm {B}} ^{*}_{{\mathrm {s}}2} \rightarrow {\mathrm {B}} ^0 \mathrm {K} ^0_{\mathrm {S}} )}{\mathcal {B}({\mathrm {B}} ^{*}_{{\mathrm {s}}2} \rightarrow {\mathrm {B}} ^+ \mathrm {K} ^- )} \nonumber \\= & {} \frac{N({\mathrm {B}} ^{*}_{{\mathrm {s}}2} \rightarrow {\mathrm {B}} ^0 \mathrm {K} ^0_{\mathrm {S}} )}{N({\mathrm {B}} ^{*}_{{\mathrm {s}}2} \rightarrow {\mathrm {B}} ^+ \mathrm {K} ^- )}\, \frac{\epsilon ({\mathrm {B}} ^{*}_{{\mathrm {s}}2} \rightarrow {\mathrm {B}} ^+ \mathrm {K} ^- )}{\epsilon ({\mathrm {B}} ^{*}_{{\mathrm {s}}2} \rightarrow {\mathrm {B}} ^0 \mathrm {K} ^0_{\mathrm {S}} )}\nonumber \\&\times \frac{\mathcal {B}({\mathrm {B}} ^+ \rightarrow {\mathrm {J}}/\psi \mathrm {K} ^+ )}{\mathcal {B}({\mathrm {B}} ^0 \rightarrow {\mathrm {J}}/\psi \mathrm {K} ^{*0} )\mathcal {B}(\mathrm {K} ^{*0} \rightarrow \mathrm {K} ^+ \pi ^- )\mathcal {B}(\mathrm {K} ^0_{\mathrm {S}} \rightarrow \pi ^+\pi ^- )}, \nonumber \\\end{aligned}$$

2 $$\begin{aligned} R^{0\pm }_{1}= & {} \frac{\mathcal {B}({\mathrm {B}} _{{\mathrm {s}}1} \rightarrow {\mathrm {B}} ^{*0} \mathrm {K} ^0_{\mathrm {S}} )}{\mathcal {B}({\mathrm {B}} _{{\mathrm {s}}1} \rightarrow {\mathrm {B}} ^{*+} \mathrm {K} ^- )} \nonumber \\= & {} \frac{N({\mathrm {B}} _{{\mathrm {s}}1} \rightarrow {\mathrm {B}} ^{*0} \mathrm {K} ^0_{\mathrm {S}} )}{N({\mathrm {B}} _{{\mathrm {s}}1} \rightarrow {\mathrm {B}} ^{*+} \mathrm {K} ^- )}\,\frac{\epsilon ({\mathrm {B}} _{{\mathrm {s}}1} \rightarrow {\mathrm {B}} ^{*+} \mathrm {K} ^- )}{\epsilon ({\mathrm {B}} _{{\mathrm {s}}1} \rightarrow {\mathrm {B}} ^{*0} \mathrm {K} ^0_{\mathrm {S}} )}\nonumber \\&\times \frac{\mathcal {B}({\mathrm {B}} ^+ \rightarrow {\mathrm {J}}/\psi \mathrm {K} ^+ )}{\mathcal {B}({\mathrm {B}} ^0 \rightarrow {\mathrm {J}}/\psi \mathrm {K} ^{*0} )\mathcal {B}(\mathrm {K} ^{*0} \rightarrow \mathrm {K} ^+ \pi ^- )\mathcal {B}(\mathrm {K} ^0_{\mathrm {S}} \rightarrow \pi ^+\pi ^- )}, \nonumber \\\end{aligned}$$

3 $$\begin{aligned} R^{\pm }_{2*}= & {} \frac{\mathcal {B}({\mathrm {B}} ^{*}_{{\mathrm {s}}2} \rightarrow {\mathrm {B}} ^{*+} \mathrm {K} ^- )}{\mathcal {B}({\mathrm {B}} ^{*}_{{\mathrm {s}}2} \rightarrow {\mathrm {B}} ^+ \mathrm {K} ^- )}\nonumber \\= & {} \frac{N({\mathrm {B}} ^{*}_{{\mathrm {s}}2} \rightarrow {\mathrm {B}} ^{*+} \mathrm {K} ^- )}{N({\mathrm {B}} ^{*}_{{\mathrm {s}}2} \rightarrow {\mathrm {B}} ^+ \mathrm {K} ^- )} \, \frac{\epsilon ({\mathrm {B}} ^{*}_{{\mathrm {s}}2} \rightarrow {\mathrm {B}} ^+ \mathrm {K} ^- )}{\epsilon ({\mathrm {B}} ^{*}_{{\mathrm {s}}2} \rightarrow {\mathrm {B}} ^{*+} \mathrm {K} ^- )}, \end{aligned}$$

4 $$\begin{aligned} R^{0}_{2*}= & {} \frac{\mathcal {B}({\mathrm {B}} ^{*}_{{\mathrm {s}}2} \rightarrow {\mathrm {B}} ^{*0} \mathrm {K} ^0_{\mathrm {S}} )}{\mathcal {B}({\mathrm {B}} ^{*}_{{\mathrm {s}}2} \rightarrow {\mathrm {B}} ^0 \mathrm {K} ^0_{\mathrm {S}} )}\nonumber \\= & {} \frac{N({\mathrm {B}} ^{*}_{{\mathrm {s}}2} \rightarrow {\mathrm {B}} ^{*0} \mathrm {K} ^0_{\mathrm {S}} )}{N({\mathrm {B}} ^{*}_{{\mathrm {s}}2} \rightarrow {\mathrm {B}} ^0 \mathrm {K} ^0_{\mathrm {S}} )} \, \frac{\epsilon ({\mathrm {B}} ^{*}_{{\mathrm {s}}2} \rightarrow {\mathrm {B}} ^0 \mathrm {K} ^0_{\mathrm {S}} )}{\epsilon ({\mathrm {B}} ^{*}_{{\mathrm {s}}2} \rightarrow {\mathrm {B}} ^{*0} \mathrm {K} ^0_{\mathrm {S}} )}, \end{aligned}$$

5 $$\begin{aligned} R^{\pm }_{\sigma }= & {} \frac{\sigma (\mathrm {pp} \rightarrow {\mathrm {B}} _{{\mathrm {s}}1} \mathrm {X})\,\mathcal {B}({\mathrm {B}} _{{\mathrm {s}}1} \rightarrow {\mathrm {B}} ^{*+} \mathrm {K} ^- )}{\sigma (\mathrm {pp} \rightarrow {\mathrm {B}} ^{*}_{{\mathrm {s}}2} \mathrm {X})\,\mathcal {B}({\mathrm {B}} ^{*}_{{\mathrm {s}}2} \rightarrow {\mathrm {B}} ^+ \mathrm {K} ^- )} \nonumber \\= & {} \frac{N({\mathrm {B}} _{{\mathrm {s}}1} \rightarrow {\mathrm {B}} ^{*+} \mathrm {K} ^- )}{N({\mathrm {B}} ^{*}_{{\mathrm {s}}2} \rightarrow {\mathrm {B}} ^+ \mathrm {K} ^- )} \, \frac{\epsilon ({\mathrm {B}} ^{*}_{{\mathrm {s}}2} \rightarrow {\mathrm {B}} ^+ \mathrm {K} ^- )}{\epsilon ({\mathrm {B}} _{{\mathrm {s}}1} \rightarrow {\mathrm {B}} ^{*+} \mathrm {K} ^- )}, \end{aligned}$$

6 $$\begin{aligned} R^{0}_{\sigma }= & {} \frac{\sigma (\mathrm {pp} \rightarrow {\mathrm {B}} _{{\mathrm {s}}1} \mathrm {X})\,\mathcal {B}({\mathrm {B}} _{{\mathrm {s}}1} \rightarrow {\mathrm {B}} ^{*0} \mathrm {K} ^0_{\mathrm {S}} )}{\sigma (\mathrm {pp} \rightarrow {\mathrm {B}} ^{*}_{{\mathrm {s}}2} \mathrm {X})\,\mathcal {B}({\mathrm {B}} ^{*}_{{\mathrm {s}}2} \rightarrow {\mathrm {B}} ^0 \mathrm {K} ^0_{\mathrm {S}} )} \nonumber \\= & {} \frac{N({\mathrm {B}} _{{\mathrm {s}}1} \rightarrow {\mathrm {B}} ^{*0} \mathrm {K} ^0_{\mathrm {S}} )}{N({\mathrm {B}} ^{*}_{{\mathrm {s}}2} \rightarrow {\mathrm {B}} ^0 \mathrm {K} ^0_{\mathrm {S}} )} \, \frac{\epsilon ({\mathrm {B}} ^{*}_{{\mathrm {s}}2} \rightarrow {\mathrm {B}} ^0 \mathrm {K} ^0_{\mathrm {S}} )}{\epsilon ({\mathrm {B}} _{{\mathrm {s}}1} \rightarrow {\mathrm {B}} ^{*0} \mathrm {K} ^0_{\mathrm {S}} )}, \end{aligned}$$

where $\mathrm {X}$ stands for an inclusive reaction, and $N(\mathrm {A}\rightarrow \mathrm {BC})$ and $\epsilon (\mathrm {A}\rightarrow \mathrm {BC})$ correspond to the number of $\mathrm {A}\rightarrow \mathrm {BC}$ decays observed in data and the total efficiency for the $\mathrm {A}\rightarrow \mathrm {BC}$ decay, respectively. The branching fractions of the decays ${\mathrm {B}} ^{*+} \rightarrow {\mathrm {B}} ^+ \gamma$ and ${\mathrm {B}} ^{*0} \rightarrow {\mathrm {B}} ^0 \gamma$ are assumed to be 100%. Additionally, the mass differences in the studied decays and the natural width of the ${\mathrm {B}} ^{*}_{{\mathrm {s}}2}(5840)^0$ state are measured, as well as the mass differences $M_{{\mathrm {B}} ^0}-M_{{\mathrm {B}} ^+}$ and $M_{{\mathrm {B}} ^{*0}}-M_{{\mathrm {B}} ^{*+}}$. The data sample corresponds to an integrated luminosity of  of proton-proton collisions at $\sqrt{s}= 8\,\text {TeV}$, collected by the CMS experiment [7] at the CERN LHC in 2012.

## The CMS detector

The central feature of the CMS apparatus is a superconducting solenoid of 6$\text {\,m}$ internal diameter, providing a magnetic field of 3.8$\text {\,T}$. Within the solenoid volume are a silicon pixel and strip tracker, a lead tungstate crystal electromagnetic calorimeter, and a brass and scintillator hadron calorimeter, each composed of a barrel and two endcap sections. Muons are detected in the pseudorapidity range $|\eta |<2.4$ in gas-ionization chambers embedded in the steel flux-return yoke outside the solenoid. The main subdetectors used for the present analysis are the silicon tracker and the muon detection system. The silicon tracker measures charged particles within the range $|\eta | < 2.5$. For nonisolated particles with transverse momentum $1< p_{\mathrm {T}} < 10\,\text {GeV}$ and $|\eta | < 1.4$, the track resolutions are typically 1.5% in $p_{\mathrm {T}}$ and 25–90 (45–150)$\,\mu \text {m}$ in the transverse (longitudinal) impact parameter [8]. Matching muons to tracks measured in the silicon tracker results in a relative $p_{\mathrm {T}}$ resolution for muons with $p_{\mathrm {T}} < 10\,\text {GeV}$ of 0.8–3.0% depending on $|\eta |$ [9]. A more detailed description of the CMS detector, together with a definition of the coordinate system used and the relevant kinematic variables, can be found in Ref. [7].

Events of interest are selected using a two-tiered trigger system [10]. The first level, composed of custom hardware processors, uses information from the calorimeters and muon detectors to select events at a rate of around 100$\text {\,kHz}$ within a time interval of less than 4$\,\mu \text {s}$. The second level, known as the high-level trigger (HLT), consists of a farm of processors running a version of the full event reconstruction software optimized for fast processing, and reduces the event rate to around 1$\text {\,kHz}$ before data storage.

## Event reconstruction and selection

The data sample is collected with an HLT algorithm designed to select events with two muons consistent with originating from a charmonium resonance decaying at a significant distance from the beam axis. The requirements imposed at the trigger level include $p_{\mathrm {T}} (\mu ^\pm ) >3.5\,\text {GeV}$, $|\eta (\mu ^\pm ) |<2.2$, $p_{\mathrm {T}} (\mu ^+\mu ^-) >6.9\,\text {GeV}$, dimuon vertex $\chi ^2$ fit probability $P_{\text {vtx}}(\mu ^+\mu ^-) > 10\%$, dimuon invariant mass $1.0<M(\mu ^+\mu ^-)<4.8\,\text {GeV}$, distance between the beam axis and the reconstructed dimuon vertex position in the transverse plane $L_{xy}(\mu ^+\mu ^-) > 3 \sigma _{L_{xy}(\mu ^+\mu ^-)}$, where $\sigma _{L_{xy}(\mu ^+\mu ^-)}$ is the uncertainty in $L_{xy}(\mu ^+\mu ^-)$, and the cosine of the dimuon candidate pointing angle to the beam axis $\cos (\vec {L}_{xy}(\mu ^+\mu ^-)$, $\vec {p_{\mathrm {T}}}(\mu ^+\mu ^-)) > 0.9$. The pointing angle is the angle between the $\mu ^+\mu ^-$ candidate momentum in the transverse (*x*–*y*) plane and the vector from the beam axis position to the reconstructed dimuon vertex in the transverse plane.

The reconstruction and selection of the ${\mathrm {B}}$ meson candidates are similar to those described in Ref. [11]. The analysis requires two muons of opposite charge that must match those that triggered the event readout. The trigger requirements are confirmed and the ${\mathrm {J}}/\psi$ candidates are selected by tightening the dimuon mass region to $[3.04,\,3.15]\,\text {GeV}$.

**Fig. 1:**
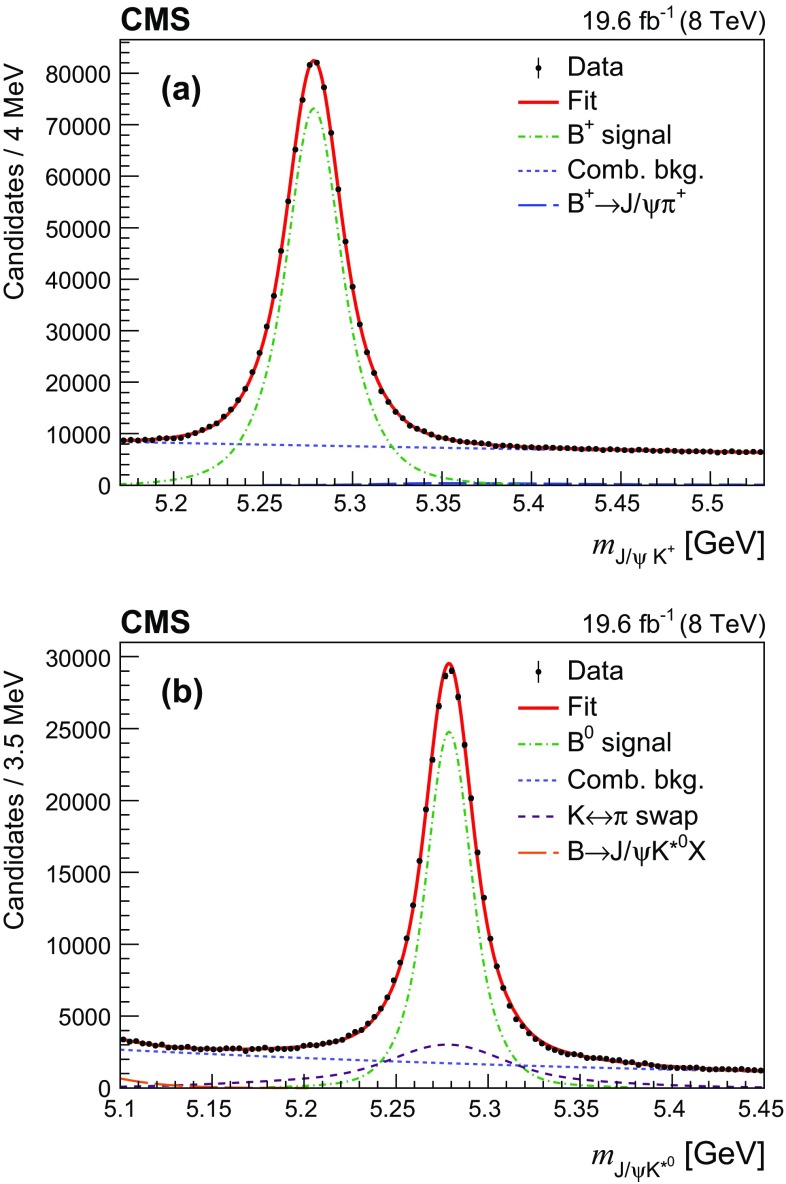
Invariant mass distributions of **a** ${\mathrm {J}}/\psi \mathrm {K} ^+$ and **b** ${\mathrm {J}}/\psi \mathrm {K} ^{*0}$ candidates in data with the fit results superimposed. The points represent the data, with the vertical bars giving the corresponding statistical uncertainties. The thick curves are results of the fits, the dash-dotted lines display the signal contributions, and the short-dashed lines show the combinatorial background contributions. The long-dashed line shows in **a** the contribution from the ${\mathrm {B}} ^+ \rightarrow {\mathrm {J}}/\psi \pi ^+$ decay, and in **b** the contribution from partially reconstructed ${\mathrm {B}} \rightarrow {\mathrm {J}}/\psi \mathrm {K} ^{*0} \mathrm {X}$ decays. The dashed line in **b** displays the contribution from swapping $\mathrm {K} ^\pm \rightarrow \pi ^\pm$ in the reconstruction

The ${\mathrm {B}} ^+ \rightarrow {\mathrm {J}}/\psi \mathrm {K} ^+$ candidates are constructed by combining the selected ${\mathrm {J}}/\psi$ candidates with a track having $p_{\mathrm {T}} >1\,\text {GeV}$ to which the kaon mass is assigned. The muon candidates must also satisfy the soft-muon identification criteria described in Ref. [9], and the kaon candidates must pass the high-purity track requirements detailed in Ref. [8]. A kinematic fit to the three tracks is performed that constrains the dimuon invariant mass to the world-average ${\mathrm {J}}/\psi$ mass [12]. From all the reconstructed $\mathrm {pp}$ collision vertices in an event, the primary vertex (PV) is chosen as the one with the smallest ${\mathrm {B}} ^+$ pointing angle. This pointing angle is the angle between the ${\mathrm {B}} ^+$ candidate momentum and the vector from the PV to the reconstructed ${\mathrm {B}} ^+$ candidate vertex. Furthermore, in this procedure, if any of the three tracks used in the ${\mathrm {B}} ^+$ candidate reconstruction are included in the fit of the chosen PV, they are removed, and the PV is refitted. The ${\mathrm {B}} ^+$ candidates are required to have $p_{\mathrm {T}} ({\mathrm {B}} ^+) >10\,\text {GeV}$, $P_{\text {vtx}}({\mathrm {B}} ^+)>1\%$, $L_{xy}({\mathrm {B}} ^+) > 5 \sigma _{L_{xy}({\mathrm {B}} ^+)}$, and $\cos (\vec {L}_{xy}({\mathrm {B}} ^+)$, $\vec {p_{\mathrm {T}}}({\mathrm {B}} ^+))> 0.99$. The invariant mass distribution of the ${\mathrm {B}} ^+ \rightarrow {\mathrm {J}}/\psi \mathrm {K} ^+$ candidates is shown in Fig. 1a. An unbinned extended maximum-likelihood fit is performed to this distribution using a triple-Gaussian function with common mean for the signal, an exponential function for the combinatorial background, and a fixed-shape function, derived from simulation, accounting for the Cabibbo-suppressed ${\mathrm {B}} ^+ \rightarrow {\mathrm {J}}/\psi \pi ^+$ decay. The parameters of the signal and the combinatorial background contributions, as well as the yields of the different components, are free in the fit. The effective resolution of the signal function ($\sigma _{M_{{\mathrm {B}} ^+}}$) found from simulation of about 24$\,\text {MeV}$ is consistent with the resolution measured in data. The invariant mass $M({\mathrm {B}} ^+)$ returned by the vertex fit is required to lie in the range $[5.23, 5.33]\,\text {GeV}$, corresponding to a $\pm 2\sigma _{M_{{\mathrm {B}} ^+}}$ window around the ${\mathrm {B}} ^+$ mass.

The selected ${\mathrm {B}} ^+$ candidates are combined with each track originating from the chosen PV with the charged kaon mass assigned to it. The track charge must be opposite to that of the reconstructed ${\mathrm {B}} ^+$ meson candidate (in the following, this track is referred to as $\mathrm {K} ^-$). The kaon candidate is required to fulfill the standard high-purity track requirements [8] and have $p_{\mathrm {T}} (\mathrm {K} ^-)>1\,\text {GeV}$.

The reconstruction of ${\mathrm {B}} ^0 \rightarrow {\mathrm {J}}/\psi (\mu ^+\mu ^-)\mathrm {K} ^{*0} (\mathrm {K} ^+ \pi ^-)$ candidates is similar to the one used for the charged decay mode. The dimuon combinations forming ${\mathrm {J}}/\psi$ candidates are obtained using the same algorithm. The ${\mathrm {B}} ^0$ candidates are constructed from the selected ${\mathrm {J}}/\psi$ candidates and two tracks of opposite charge, assumed to be from a kaon and a pion. The tracks are required to satisfy standard high-purity track requirements [8] and have $p_{\mathrm {T}} >1\,\text {GeV}$. Those kaon and pion candidates that can be matched to a signal in the muon chambers are rejected.

The ${\mathrm {B}} ^0$ candidates are obtained by performing a kinematic vertex fit to the four tracks described above that constrains the dimuon invariant mass to that of the ${\mathrm {J}}/\psi$ meson [12]. The candidates are required to have $L_{xy}({\mathrm {B}} ^0) > 5 \sigma _{L_{xy}({\mathrm {B}} ^0)}$, $P_{\text {vtx}}(\mu ^+\mu ^- \mathrm {K} ^+ \pi ^-)>1\%$, $\cos (\vec {L}_{xy}({\mathrm {B}} ^0),\vec {p_{\mathrm {T}}}({\mathrm {B}} ^0))> 0.99$, and $p_{\mathrm {T}} ({\mathrm {B}} ^0) >10\,\text {GeV}$. To reject the contribution from ${\mathrm {B}} ^0_{\mathrm {s}} \rightarrow {\mathrm {J}}/\psi \phi$ decay, the invariant mass of the two hadron tracks, if both are assigned the kaon mass, is required to be above 1.035$\,\text {GeV}$. We demand that the $\mathrm {K} ^+ \pi ^-$ invariant mass is within 90$\,\text {MeV}$ of the $\mathrm {K} ^{*0}$ mass [12]. If both the $\mathrm {K} ^+ \pi ^-$ and $\mathrm {K} ^- \pi ^+$ hypotheses pass this selection, then the $\mathrm {K} ^+ \pi ^-$ invariant mass must be closer to the $\mathrm {K} ^{*0}$ mass than the $\mathrm {K} ^- \pi ^+$ invariant mass. The invariant mass distribution of the selected ${\mathrm {B}} ^0 \rightarrow {\mathrm {J}}/\psi \mathrm {K} ^+ \pi ^-$ candidates is shown in Fig. 1b. It is fitted with a sum of a triple-Gaussian function with a common mean for the signal, a double-Gaussian function accounting for the $\mathrm {K} ^\pm \rightarrow \pi ^\pm$ swapped (KPS) component, where the second Gaussian is asymmetric, and an exponential function for the combinatorial background. An additional Gaussian function is included to account for the partially reconstructed ${\mathrm {B}} \rightarrow {\mathrm {J}}/\psi \mathrm {K} ^{*0} \mathrm {X}$ background near the left edge of the fit region. The resolution parameters of the signal function and the parameters of the KPS are fixed to the values obtained in simulation; the other parameters are free in the fit. The effective resolution of the signal function ($\sigma _{M_{{\mathrm {B}} ^0}}$) found from the simulation is about 19$\,\text {MeV}$. The ${\mathrm {B}} ^0$ candidate returned by the vertex fit is required to have an invariant mass in the range 5.245 to 5.313$\,\text {GeV}$, corresponding to approximately $\pm 2\sigma _{M_{{\mathrm {B}} ^0}}$ around the known ${\mathrm {B}} ^0$ mass [12]. The fit results are used to extract the fraction of the KPS with respect to the signal yield in the ${\mathrm {B}} ^0$ signal region of $(18.9\pm 0.3)\%$, where the uncertainty is statistical only.

The selected ${\mathrm {B}} ^0$ candidates are combined with $\mathrm {K} ^0_{\mathrm {S}}$ candidates that are formed from detached two-prong vertices, assuming the decay $\mathrm {K} ^0_{\mathrm {S}} \rightarrow \pi ^+\pi ^-$, as described in Ref. [13]. The two-pion invariant mass is required to be within $\pm 20\,\text {MeV}$ of the $\mathrm {K} ^0_{\mathrm {S}}$ mass [12], which corresponds approximately to 4 times the $\pi ^+\pi ^-$ mass resolution. The two pion tracks are refitted with their invariant mass constrained to the known $\mathrm {K} ^0_{\mathrm {S}}$ mass, and the obtained $\mathrm {K} ^0_{\mathrm {S}}$ candidate is required to satisfy $P_{\text {vtx}}(\mathrm {K} ^0_{\mathrm {S}})>1\%$ and $\cos (\vec {L}_{xy}(\mathrm {K} ^0_{\mathrm {S}}),\vec {p_{\mathrm {T}}}(\mathrm {K} ^0_{\mathrm {S}}))> 0.999$. Multiple candidates from the same event are not removed.

Simulated events that are used to obtain relative efficiencies and invariant mass resolutions are produced with pythia v6.424 [14]. The ${\mathrm {b}}$ hadron decays are modelled with evtgen 1.3.0 [15]. Final-state photon radiation is included in evtgen using photos  [16, 17]. The events are then passed through a detailed Geant4-based simulation [18] of the CMS detector with the same trigger and reconstruction algorithms as used for the data. The simulation includes effects from multiple $\mathrm {pp}$ interactions in the same or nearby beam crossings (pileup) with the same multiplicity distribution as observed in data. Matching of the reconstructed candidates to the generated particles is obtained by requiring $\varDelta R=\sqrt{\smash [b]{(\varDelta \eta )^2 + (\varDelta \phi )^2}}$ to be <0.015 for $\pi ^\pm$ and $\mathrm {K} ^\pm$, <0.004 for muons, and <0.020 for $\mathrm {K} ^0_{\mathrm {S}}$, where $\varDelta \eta$ and $\varDelta \phi$ are the differences in pseudorapidity and azimuthal angle (in radians), respectively, between the three-momenta of the reconstructed and generated particles.

## Fits to the ${\mathrm {B}} \mathrm {K}$ invariant mass distributions

For every invariant mass distribution fit discussed in this section, the functional models for the signal and the combinatorial background components are chosen such that a good description of the binned distribution is obtained. The description quality is verified using the difference between the data and fit result, divided by the statistical uncertainty in the data and also with $\chi ^2$ tests.

### ${\mathrm {B}} ^+ \mathrm {K} ^-$ invariant mass

To improve the ${\mathrm {B}} ^+ \mathrm {K} ^-$ invariant mass resolution, the variable $m_{{\mathrm {B}} ^+ \mathrm {K} ^-}$ is computed as

$$\begin{aligned} m_{{\mathrm {B}} ^+ \mathrm {K} ^-} = M({\mathrm {B}} ^+ \mathrm {K} ^-) - M({\mathrm {B}} ^+) + M_{{\mathrm {B}} ^+}^{\mathrm {PDG}}, \end{aligned}$$

where $M({\mathrm {B}} ^+ \mathrm {K} ^-)$ is the invariant mass of the reconstructed ${\mathrm {B}} ^+ \mathrm {K} ^-$ combination, $M({\mathrm {B}} ^+)$ is the reconstructed ${\mathrm {B}} ^+$ mass, and $M_{{\mathrm {B}} ^+}^{\mathrm {PDG}}$ is the world-average ${\mathrm {B}} ^+$ meson mass [12].

**Fig. 2:**
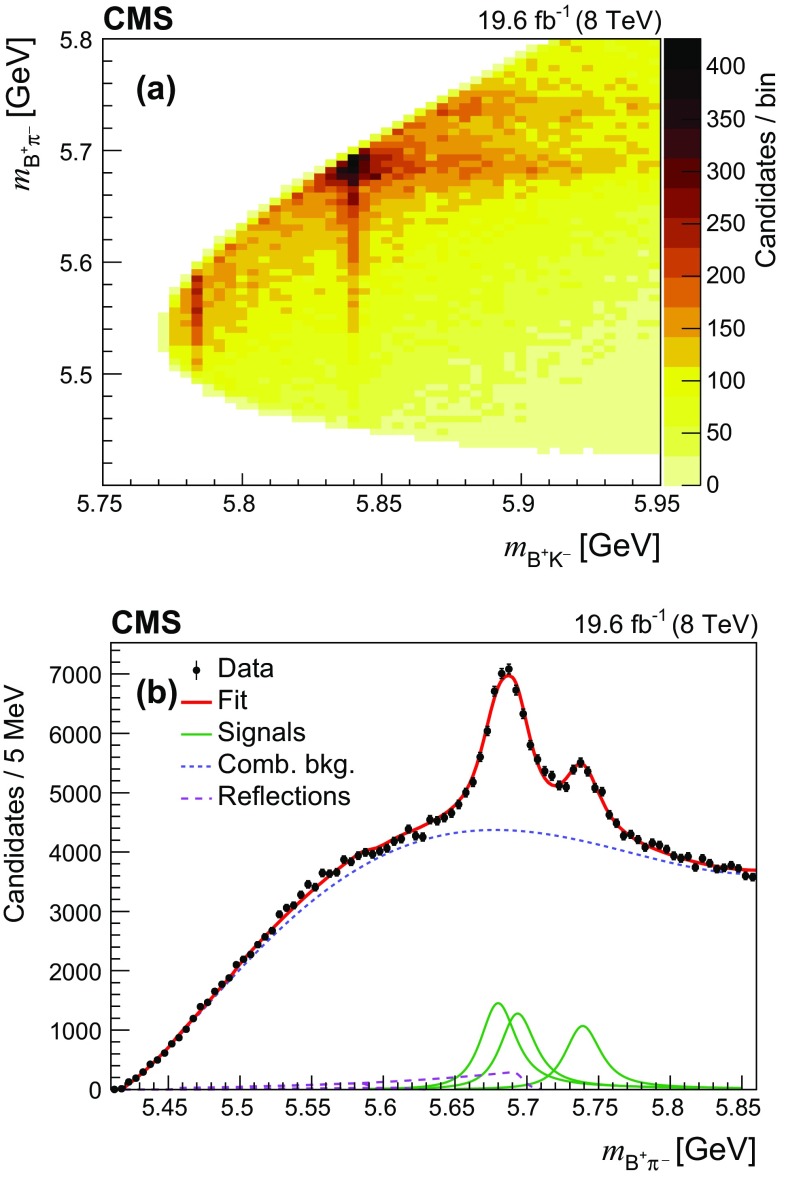
**a** Two-dimensional distribution of $m_{{\mathrm {B}} ^+ \mathrm {K} ^-}$ versus $m_{{\mathrm {B}} ^+ \pi ^-}$ in data. **b** The fitted ${\mathrm {B}} ^+ \pi ^-$ invariant mass distribution. The points represent the data, the thick solid curve is the fit projection, the thin lines indicate the three excited ${\mathrm {B}} ^0$ signal contributions, the short-dashed curve is the combinatorial background, and the long-dashed lines show the contributions from the excited ${\mathrm {B}} ^0_{\mathrm {s}}$ decays

The decays of excited ${\mathrm {B}} ^0$ mesons ${\mathrm {B}} _{1} \rightarrow {\mathrm {B}} ^{*+} \pi ^-$, ${\mathrm {B}} ^{*}_{2} \rightarrow {\mathrm {B}} ^+ \pi ^-$, and ${\mathrm {B}} ^{*}_{2} \rightarrow {\mathrm {B}} ^{*+} \pi ^-$ contribute to the obtained ${\mathrm {B}} ^+ \mathrm {K} ^-$ mass distribution, as seen from the two-dimensional distribution in Fig. 2a. It is important to take into account these background contributions in the fits to the $m_{{\mathrm {B}} ^+ \mathrm {K} ^-}$ distribution. Simulated samples of these decays are reconstructed in the same way as the collision events to obtain the corresponding reflection shapes in the $m_{{\mathrm {B}} ^+ \mathrm {K} ^-}$ distribution. In order to measure the yields of these reflections, the ${\mathrm {B}} ^+ \pi ^-$ invariant mass, $m_{{\mathrm {B}} ^+ \pi ^-}$, is computed the same way as $m_{{\mathrm {B}} ^+ \mathrm {K} ^-}$. Fits are performed on the $m_{{\mathrm {B}} ^+ \pi ^-}$ distribution observed in data, using the same data set, with a pion mass assigned to the track instead of a kaon mass. Then the obtained yields of these contributions are used in the fits to the $m_{{\mathrm {B}} ^+ \mathrm {K} ^-}$ distribution.

The measured $m_{{\mathrm {B}} ^+ \pi ^-}$ distribution is presented in Fig. 2b. Clear enhancements are seen around 5.65–5.75$\,\text {GeV}$, corresponding to the decays of excited ${\mathrm {B}} ^0$ mesons. An unbinned extended maximum-likelihood fit is performed to this distribution. The three signal functions accounting for the ${\mathrm {B}} ^{*}_{2} \rightarrow {\mathrm {B}} ^+ \pi ^-$, ${\mathrm {B}} ^{*}_{2} \rightarrow {\mathrm {B}} ^{*+} \pi ^-$, and ${\mathrm {B}} _{1} \rightarrow {\mathrm {B}} ^{*+} \pi ^-$ decays are *D*-wave relativistic Breit–Wigner (RBW) functions, convolved with a double-Gaussian resolution function, with parameters fixed according to the simulation (the typical effective resolution is about 5.5$\,\text {MeV}$, significantly below the natural widths of the states). As verified in simulations, the signal shapes of ${\mathrm {B}} ^{*}_{2} \rightarrow {\mathrm {B}} ^{*+} \pi ^-$ and ${\mathrm {B}} _{1} \rightarrow {\mathrm {B}} ^{*+} \pi ^-$ decays (where the photon from the ${\mathrm {B}} ^{*+}$ decay is lost and only the ${\mathrm {B}} ^+ \pi ^-$ mass is reconstructed) are simply shifted by the mass difference $M_{{\mathrm {B}} ^{*+}}^{\mathrm {PDG}}-M_{{\mathrm {B}} ^+}^{\mathrm {PDG}} =45.34\pm 0.23\,\text {MeV}$ [12]. The combinatorial background is parametrized by the function $(x-x_0)^{\alpha }\, P_n(x)$, where $x\equiv m_{{\mathrm {B}} ^+ \pi ^-}$, $x_0$ is the threshold value, $\alpha$ is a free parameter, and $P_n$ is a polynomial of degree *n*, with $n=3$. Additional, relatively small contributions come from the excited ${\mathrm {B}} ^0_{\mathrm {s}}$ decays. They are included in the fit with free normalizations and fixed shapes, obtained from the simulation.

In the nominal fit, the masses and natural widths of the excited ${\mathrm {B}} ^0$ mesons are fixed to their world-average values [12]. The fit region is not extended to values above 5865$\,\text {MeV}$ to avoid having to model the ${\mathrm {B}} (5970)$ contribution [6]. The fitted event yields are about 8500, 10 500, and 12 000 for the ${\mathrm {B}} ^{*}_{2} \rightarrow {\mathrm {B}} ^+ \pi ^-$, ${\mathrm {B}} ^{*}_{2} \rightarrow {\mathrm {B}} ^{*+} \pi ^-$, and ${\mathrm {B}} _{1} \rightarrow {\mathrm {B}} ^{*+} \pi ^-$ signals, respectively.

**Fig. 3:**
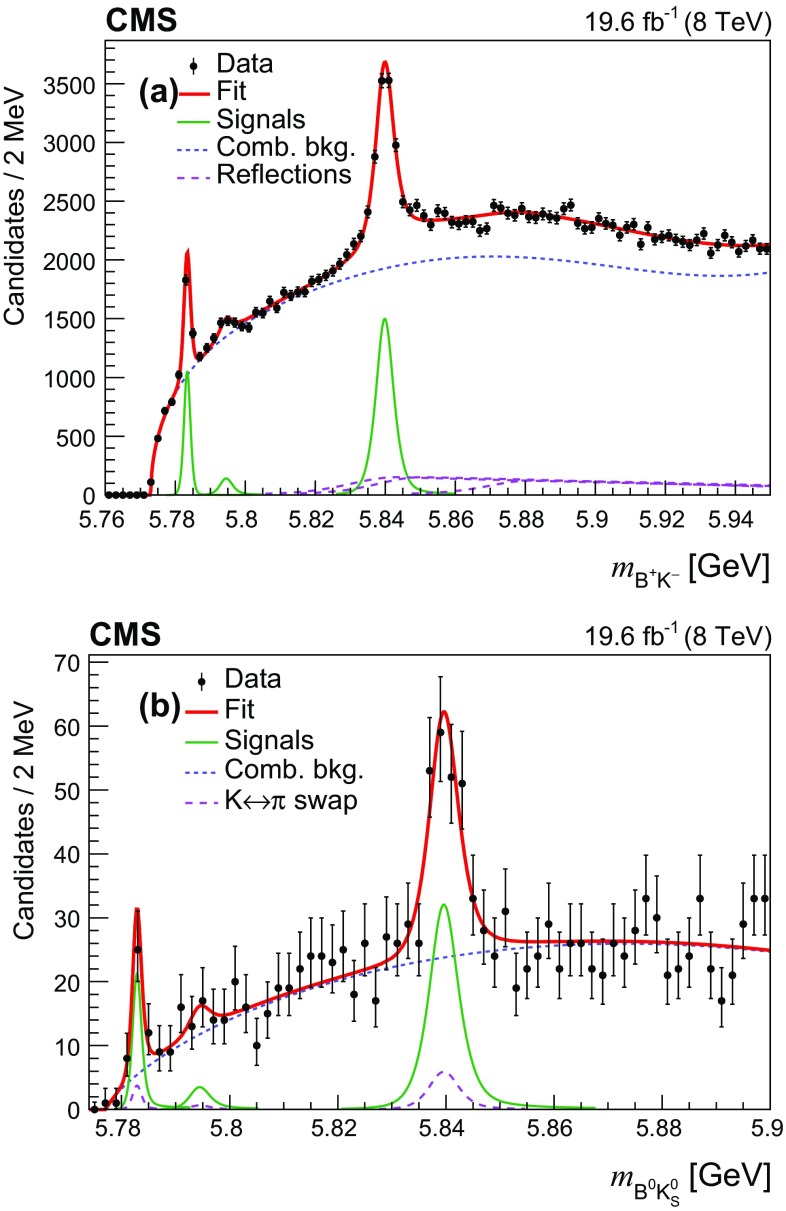
Invariant mass distributions of **a** ${\mathrm {B}} ^+ \mathrm {K} ^-$ and **b** ${\mathrm {B}} ^0 \mathrm {K} ^0_{\mathrm {S}}$ candidates with the results of the fit overlaid. The points represent the data, the thick solid curves are the results of the overall fits, and the thin solid lines display the signal contributions. The short-dashed lines show the combinatorial background contributions. The long-dashed lines show: in **a** the contributions from excited ${\mathrm {B}} ^0$ meson decays, and in **b** the contributions from swapping $\mathrm {K} ^\pm \rightarrow \pi ^\pm$ in the reconstruction of the ${\mathrm {B}} ^0$ mesons

Figure 3a shows the measured $m_{{\mathrm {B}} ^+ \mathrm {K} ^-}$ distribution. The three peaks from lower to higher mass correspond to the decays ${\mathrm {B}} _{{\mathrm {s}}1} \rightarrow {\mathrm {B}} ^{*+} \mathrm {K} ^-$, ${\mathrm {B}} ^{*}_{{\mathrm {s}}2} \rightarrow {\mathrm {B}} ^{*+} \mathrm {K} ^-$, and ${\mathrm {B}} ^{*}_{{\mathrm {s}}2} \rightarrow {\mathrm {B}} ^+ \mathrm {K} ^-$. An unbinned extended maximum-likelihood fit is performed to this distribution using the sum of three signal functions, a background function, and the three reflections from the excited ${\mathrm {B}} ^0$ decays. The signals are described with *D*-wave RBW functions convolved with double-Gaussian resolution functions obtained from the simulation (the effective resolutions are about 1–2$\,\text {MeV}$). The natural widths of the ${\mathrm {B}} ^{(*)}_{{\mathrm {s}}1,2}$ states and their masses are free parameters in the fit. The nonresonant background is modelled by $(x-x_0)^{\alpha }\, P_n(x)$, where $x\equiv m_{{\mathrm {B}} ^+ \mathrm {K} ^-}$, $x_0$ is the threshold value, and the nominal fit uses $n=6$. The reflections correspond to the contributions of excited ${\mathrm {B}} ^0$ meson decays into a ${\mathrm {B}} ^{(*)+}$ meson and a charged pion, as described above. The shapes of these contributions are obtained from the simulation and are fixed in the fit to the data. The yields of these reflections are corrected by the efficiency of using the restricted fit region $x_0<m_{{\mathrm {B}} ^+ \mathrm {K} ^-} <5.95\,\text {GeV}$. The results of the fit are presented in the second column of Table 2, where the measured masses of the ${\mathrm {B}} ^{*}_{{\mathrm {s}}2}$ and ${\mathrm {B}} _{{\mathrm {s}}1}$ mesons are given with respect to the corresponding world-average ${\mathrm {B}} ^+$ or ${\mathrm {B}} ^{*+}$, and $\mathrm {K} ^-$ masses [12].

**Table 2:** The observed signal yields (*N*), natural widths ($\varGamma$), and mass differences from the fits to the $m_{{\mathrm {B}} \mathrm {K} }$ distributions in data. The uncertainties are statistical only

	${\mathrm {B}} ^+ \mathrm {K} ^-$	${\mathrm {B}} ^0 \mathrm {K} ^0_{\mathrm {S}}$
$N({\mathrm {B}} ^{*}_{{\mathrm {s}}2} \rightarrow {\mathrm {B}} \mathrm {K})$	$5424\pm 269$	$128\pm 22$
$N({\mathrm {B}} ^{*}_{{\mathrm {s}}2} \rightarrow {\mathrm {B}} ^{*} \mathrm {K})$	$455\pm 119$	$12\pm 11$
$N({\mathrm {B}} _{{\mathrm {s}}1} \rightarrow {\mathrm {B}} ^{*} \mathrm {K})$	$1329\pm 83$	$34.5\pm 8.3$
$\varGamma ({\mathrm {B}} ^{*}_{{\mathrm {s}}2})\,[\,\text {MeV} ]$	$1.52\pm 0.34$	$2.1\pm 1.3$
$\varGamma ({\mathrm {B}} _{{\mathrm {s}}1})\,[\,\text {MeV} ]$	$0.10\pm 0.15$	$0.4\pm 0.4$
$M({\mathrm {B}} ^{*}_{{\mathrm {s}}2})-M_{{\mathrm {B}}}^{\mathrm {PDG}}-M_{\mathrm {K}}^{\mathrm {PDG}} \,[\,\text {MeV} ]$	$66.93\pm 0.09$	$62.42\pm 0.48$
$M({\mathrm {B}} _{{\mathrm {s}}1})-M_{{\mathrm {B}} ^{*}}^{\mathrm {PDG}}-M_{\mathrm {K}}^{\mathrm {PDG}} \,[\,\text {MeV} ]$	$10.50\pm 0.09$	$5.65\pm 0.23$

### ${\mathrm {B}} ^0 \mathrm {K} ^0_{\mathrm {S}}$ invariant mass 

Similarly to the ${\mathrm {B}} ^+ \mathrm {K} ^-$ channel, the variable $m_{{\mathrm {B}} ^0 \mathrm {K} ^0_{\mathrm {S}}} = M({\mathrm {B}} ^0 \mathrm {K} ^0_{\mathrm {S}}) - M({\mathrm {B}} ^0) + M_{{\mathrm {B}} ^0}^{\mathrm {PDG}}$ is used for the ${\mathrm {B}} ^0 \mathrm {K} ^0_{\mathrm {S}}$ invariant mass. The $m_{{\mathrm {B}} ^0 \mathrm {K} ^0_{\mathrm {S}}}$ distribution of the selected ${\mathrm {B}} ^0 \mathrm {K} ^0_{\mathrm {S}}$ candidates is shown in Fig. 3b. There is a significant peak at about 5840$\,\text {MeV}$ and a smaller one at 5781$\,\text {MeV}$, corresponding to the decays ${\mathrm {B}} ^{*}_{{\mathrm {s}}2} \rightarrow {\mathrm {B}} ^0 \mathrm {K} ^0_{\mathrm {S}}$ and ${\mathrm {B}} _{{\mathrm {s}}1} \rightarrow {\mathrm {B}} ^{*0} \mathrm {K} ^0_{\mathrm {S}}$, respectively. The contribution from the ${\mathrm {B}} ^{*}_{{\mathrm {s}}2} \rightarrow {\mathrm {B}} ^{*0} \mathrm {K} ^0_{\mathrm {S}}$ decay, also shown in Fig. 3b at 5795$\,\text {MeV}$, is not statistically significant. However, it is still included in the fit model described below.

The decays ${\mathrm {B}} ^{*}_{{\mathrm {s}}2} \rightarrow {\mathrm {B}} ^0 \mathrm {K} ^0_{\mathrm {S}}$, ${\mathrm {B}} ^{*}_{{\mathrm {s}}2} \rightarrow {\mathrm {B}} ^{*0} \mathrm {K} ^0_{\mathrm {S}}$, and ${\mathrm {B}} _{{\mathrm {s}}1} \rightarrow {\mathrm {B}} ^{*0} \mathrm {K} ^0_{\mathrm {S}}$ are modelled using three *D*-wave RBW functions convolved with double-Gaussian resolution functions whose parameters are fixed according to the simulation. The masses and natural widths are free parameters in the fit. Similarly to the ${\mathrm {B}} ^+ \mathrm {K} ^-$ final state, if the photon from ${\mathrm {B}} ^{*0}$ decay is lost and only the ${\mathrm {B}} ^0 \mathrm {K} ^0_{\mathrm {S}}$ mass is reconstructed, the peak position is simply shifted by the mass difference $M_{{\mathrm {B}} ^{*0}}^{\mathrm {PDG}}-M_{{\mathrm {B}} ^0}^{\mathrm {PDG}} =45.18\pm 0.23\,\text {MeV}$ [12]. Studies on simulated events show that when the kaon and the pion from the ${\mathrm {B}} ^0 \rightarrow {\mathrm {J}}/\psi \mathrm {K} ^+ \pi ^-$ decay are exchanged, the three decays mentioned above produce narrow peaks at the same mass values as the signal peaks. In order to account for these KPS contributions, three additional RBW functions, convolved with double-Gaussian shapes, are added, where the parameters of these Gaussians are fixed to the values obtained in the simulation and the yields are fixed relative to the signal yields using the mistagging probability found in the fit to the ${\mathrm {B}} ^0$ invariant mass distribution. A function of the form $(x-x_0)^{\alpha }\, P_n(x)$ is used to describe the combinatorial background, where $x\equiv m_{{\mathrm {B}} ^0 \mathrm {K} ^0_{\mathrm {S}}}$, $x_0$ is the threshold value, and $n=1$. The results of the fit are presented in the last column of Table 2, where the signal yields do not include the KPS component.

The significance of the ${\mathrm {B}} ^{*}_{{\mathrm {s}}2} \rightarrow {\mathrm {B}} ^0 \mathrm {K} ^0_{\mathrm {S}}$ decay is estimated to be 6.3 standard deviations in the baseline fit model using a ratio of the fit likelihoods with and without the signal component [19]. Systematic uncertainties, discussed in the next section, are taken into account using nuisance parameters for the mass resolution, the KPS fraction, and the ${\mathrm {B}} ^{*}_{{\mathrm {s}}2}$ mass and natural width. These parameters are allowed to vary in the fits but are constrained by Gaussian probability density functions. In particular for the ${\mathrm {B}} ^{*}_{{\mathrm {s}}2}$ mass and natural width, the world-average values and their uncertainties [12] are used. Under variations of the fit range and background model, the significance varies from 6.3 to 7.0 standard deviations. Similarly, the statistical significance of the ${\mathrm {B}} _{{\mathrm {s}}1} \rightarrow {\mathrm {B}} ^{*0} \mathrm {K} ^0_{\mathrm {S}}$ signal peak is 3.9 standard deviations, where the systematic uncertainties due to the mass resolution and KPS fraction are taken into account, as well as the uncertainties in the ${\mathrm {B}} _{{\mathrm {s}}1}$ mass and natural width. The significance varies from 3.6 to 3.9 standard deviations under variations of the fit region and the background model.

## Efficiencies and systematic uncertainties

The efficiency for each decay channel is calculated using simulated signal samples. It is defined as the number of reconstructed signal events from the simulation divided by the number of generated events. The efficiency includes the detector acceptance, trigger, and candidate reconstruction efficiencies. Only the ratios of such efficiencies for different decay modes are needed in formulae (1)–(6), which reduces the systematic uncertainties in those ratios. The resulting efficiency ratios used in the measurements of the ratios of the branching fractions are:

$$\begin{aligned} \frac{\epsilon ({\mathrm {B}} ^{*}_{{\mathrm {s}}2} \rightarrow {\mathrm {B}} ^+ \mathrm {K} ^- )}{\epsilon ({\mathrm {B}} ^{*}_{{\mathrm {s}}2} \rightarrow {\mathrm {B}} ^0 \mathrm {K} ^0_{\mathrm {S}} )}&= 15.77\pm 0.18,\\ \frac{\epsilon ({\mathrm {B}} _{{\mathrm {s}}1} \rightarrow {\mathrm {B}} ^{*+} \mathrm {K} ^- )}{\epsilon ({\mathrm {B}} _{{\mathrm {s}}1} \rightarrow {\mathrm {B}} ^{*0} \mathrm {K} ^0_{\mathrm {S}} )}&= 16.33\pm 0.20,\\ \frac{\epsilon ({\mathrm {B}} ^{*}_{{\mathrm {s}}2} \rightarrow {\mathrm {B}} ^+ \mathrm {K} ^- )}{\epsilon ({\mathrm {B}} ^{*}_{{\mathrm {s}}2} \rightarrow {\mathrm {B}} ^{*+} \mathrm {K} ^- )}&= 0.961\pm 0.010,\\ \frac{\epsilon ({\mathrm {B}} ^{*}_{{\mathrm {s}}2} \rightarrow {\mathrm {B}} ^0 \mathrm {K} ^0_{\mathrm {S}} )}{\epsilon ({\mathrm {B}} ^{*}_{{\mathrm {s}}2} \rightarrow {\mathrm {B}} ^{*0} \mathrm {K} ^0_{\mathrm {S}} )}&= 0.970\pm 0.012,\\ \frac{\epsilon ({\mathrm {B}} ^{*}_{{\mathrm {s}}2} \rightarrow {\mathrm {B}} ^+ \mathrm {K} ^- )}{\epsilon ({\mathrm {B}} _{{\mathrm {s}}1} \rightarrow {\mathrm {B}} ^{*+} \mathrm {K} ^- )}&= 0.953\pm 0.010,\\ \frac{\epsilon ({\mathrm {B}} ^{*}_{{\mathrm {s}}2} \rightarrow {\mathrm {B}} ^0 \mathrm {K} ^0_{\mathrm {S}} )}{\epsilon ({\mathrm {B}} _{{\mathrm {s}}1} \rightarrow {\mathrm {B}} ^{*0} \mathrm {K} ^0_{\mathrm {S}} )}&= 0.987\pm 0.012, \end{aligned}$$

where the uncertainties are statistical only and related to the finite size of the simulated samples.

The ratios $$R^{0\pm }_{2} $$ and $$R^{0\pm }_{1} $$ involve different numbers of final-state tracks from the decay processes in the numerator and denominator, and the related signal yields are extracted from fits to different invariant mass distributions, unlike the ratios $$R^{\pm }_{2*} $$, $$R^{0}_{2*} $$, $$R^{\pm }_{\sigma } $$, and $$R^{0}_{\sigma } $$. Therefore, the systematic uncertainties are described separately for the two cases in the next two subsections.

The statistical uncertainties in the efficiency ratios are considered as sources of systematic uncertainty in the measured branching fraction ratios. The systematic uncertainties related to muon reconstruction and identification and trigger efficiencies cancel out in the ratios. Systematic uncertainties associated with the track reconstruction efficiency are assigned only in ratios involving final states with a different number of tracks. Validation studies of the simulated signal samples are performed by comparing distributions of variables employed in the event selection between simulation and background-subtracted data, using the channels with the larger yields in data (${\mathrm {B}} ^{*}_{{\mathrm {s}}2} \rightarrow {\mathrm {B}} ^+ \mathrm {K} ^-$, ${\mathrm {B}} _{{\mathrm {s}}1} \rightarrow {\mathrm {B}} ^{*+} \mathrm {K} ^-$, and ${\mathrm {B}} ^{*}_{{\mathrm {s}}2} \rightarrow {\mathrm {B}} ^0 \mathrm {K} ^0_{\mathrm {S}}$). No significant deviations are found, and no additional systematic uncertainties in the efficiency ratios are assigned.

### Systematic uncertainties in the ratios $R^{0\pm }_{2}$ and $R^{0\pm }_{1}$

A systematic uncertainty of $2\times 3.9\%=7.8\%$ [8] is assigned to the $R^{0\pm }_{2}$ and $R^{0\pm }_{1}$ ratios due to the uncertainty in the track reconstruction efficiency, since the neutral decay channel has two additional charged particles in the final state in comparison to the charged decay channel.

To evaluate the systematic uncertainties related to the choice of the invariant mass fit model, several alternative functions are tested. The systematic uncertainty in each signal yield is calculated as the highest deviation of the observed signal yield from the baseline fit result. Changes in each fit involve variations in the polynomial degree *n* in the background model and the fit range; for the fit to the $m_{{\mathrm {B}} ^+ \pi ^-}$ distribution the variations also include letting the signal masses and natural widths float. The uncertainties related to fits to the ${\mathrm {B}} ^+ \pi ^-$, ${\mathrm {B}} ^+ \mathrm {K} ^-$, and ${\mathrm {B}} ^0 \mathrm {K} ^0_{\mathrm {S}}$ invariant mass distributions are treated separately and include:

- A systematic uncertainty related to the fit to ${\mathrm {B}} ^+ \pi ^-$ invariant mass of 2.5% for $N({\mathrm {B}} ^{*}_{{\mathrm {s}}2} \rightarrow {\mathrm {B}} ^+ \mathrm {K} ^-)$ and 2.0% for $N({\mathrm {B}} _{{\mathrm {s}}1} \rightarrow {\mathrm {B}} ^{*+} \mathrm {K} ^-)$,
- A systematic uncertainty related to the fit to ${\mathrm {B}} ^+ \mathrm {K} ^-$ invariant mass of 2.4% for $N({\mathrm {B}} ^{*}_{{\mathrm {s}}2} \rightarrow {\mathrm {B}} ^+ \mathrm {K} ^-)$ and 4.6% for $N({\mathrm {B}} _{{\mathrm {s}}1} \rightarrow {\mathrm {B}} ^{*+} \mathrm {K} ^-)$,
- A systematic uncertainty related to the fit to ${\mathrm {B}} ^0 \mathrm {K} ^0_{\mathrm {S}}$ invariant mass of 14% for $N({\mathrm {B}} ^{*}_{{\mathrm {s}}2} \rightarrow {\mathrm {B}} ^0 \mathrm {K} ^0_{\mathrm {S}} )$ and 8.1% for $N({\mathrm {B}} _{{\mathrm {s}}1} \rightarrow {\mathrm {B}} ^{*0} \mathrm {K} ^0_{\mathrm {S}} )$.

The uncertainty from the invariant mass resolution is estimated by comparing the ${\mathrm {B}} ^+ \rightarrow {\mathrm {J}}/\psi \mathrm {K} ^+$ decays in data and simulation, yielding a difference of at most 2.6%. To account for this, the signal fits to the $m_{{\mathrm {B}} ^+ \mathrm {K} ^-}$ and $m_{{\mathrm {B}} ^0 \mathrm {K} ^0_{\mathrm {S}}}$ distributions in data are repeated with the resolutions decreased and increased by 3%. The largest deviations from the baseline in the measured ratios are: 0.7% for $N({\mathrm {B}} ^{*}_{{\mathrm {s}}2} \rightarrow {\mathrm {B}} ^0 \mathrm {K} ^0_{\mathrm {S}} )/N({\mathrm {B}} ^{*}_{{\mathrm {s}}2} \rightarrow {\mathrm {B}} ^+ \mathrm {K} ^- )$ and 2.2% for $N({\mathrm {B}} _{{\mathrm {s}}1} \rightarrow {\mathrm {B}} ^{*0} \mathrm {K} ^0_{\mathrm {S}} )/N({\mathrm {B}} _{{\mathrm {s}}1} \rightarrow {\mathrm {B}} ^{*+} \mathrm {K} ^- )$. These values are used as systematic uncertainties in the ratios $R^{0\pm }_{2}$ and $R^{0\pm }_{1}$.

The fraction of the KPS component in the $${\mathrm {B}} ^0 \mathrm {K} ^0_{\mathrm {S}} $$ signals is obtained from the fit to the $${\mathrm {B}} ^0 $$ invariant mass distribution in the data. The systematic uncertainty in this fraction is evaluated by varying the $${\mathrm {B}} ^0 $$ signal mass resolution by $$\pm 3\%$$. The resulting variations of the KPS fraction are at most 3%. The other variations in the fit to the $${\mathrm {J}}/\psi \mathrm {K} ^{*0} $$ invariant mass distribution result in negligible changes in the KPS fraction. The corresponding systematic uncertainty is 2.6% in both $$R^{0\pm }_{2} $$ and $$R^{0\pm }_{1} $$. As expected, the changes of the other ratios ($$R^{0}_{2*} $$, $$R^{0}_{\sigma } $$) under these variations are negligible.

Formulae (1) and (2) assume the decay ${\mathrm {B}} ^0 \rightarrow {\mathrm {J}}/\psi \mathrm {K} ^+ \pi ^-$ proceeds only through the $\mathrm {K} ^{*0}$ resonance. The systematic uncertainty related to this assumption is estimated by fitting the $\mathrm {K} ^+ \pi ^-$ invariant mass distribution obtained from the candidate ${\mathrm {B}} ^0$ data events using the background-subtraction technique ${}_\mathrm {s}$Plot [20]. This gives an estimate of 5% for the nonresonant $\mathrm {K} ^+ \pi ^-$ fraction in the total number of signal events, which is included as a systematic uncertainty in the ratios $R^{0\pm }_{2}$ and $R^{0\pm }_{1}$.

All these systematic uncertainties are summarized in Table 3, along with the total systematic uncertainty, calculated as the sum in quadrature of the different sources.

**Table 3:** Relative systematic uncertainties in percent in the ratios $R^{0\pm }_{2}$ and $R^{0\pm }_{1}$

Source	$R^{0\pm }_{2}$	$R^{0\pm }_{1}$
Track reconstruction efficiency	7.8	7.8
$m_{{\mathrm {B}} ^+ \pi ^-}$ distribution model	2.5	2.0
$m_{{\mathrm {B}} ^+ \mathrm {K} ^-}$ distribution model	2.4	4.6
$m_{{\mathrm {B}} ^0 \mathrm {K} ^0_{\mathrm {S}}}$ distribution model	14	8.1
Mass resolution	0.7	2.2
Fraction of KPS	2.6	2.6
Non-$\mathrm {K} ^{*0}$ contribution	5.0	5.0
Finite size of simulated samples	1.2	1.2
Total	18	14

**Table 4:** Relative systematic uncertainties in percent in the ratios $$R^{\pm }_{2*} $$, $$R^{0}_{2*} $$, $$R^{\pm }_{\sigma } $$, and $$R^{0}_{\sigma } $$

Source	$$R^{\pm }_{2*} $$	$$R^{0}_{2*} $$	$$R^{\pm }_{\sigma } $$	$$R^{0}_{\sigma } $$
$m_{{\mathrm {B}} ^+ \pi ^-}$ distribution model	2.9	–	2.7	–
$m_{{\mathrm {B}} ^+ \mathrm {K} ^-}$ distribution model	17	–	7.1	–
$m_{{\mathrm {B}} ^0 \mathrm {K} ^0_{\mathrm {S}}}$ distribution model	–	13	–	24
Mass resolution	1.2	3.0	1.5	1.1
Uncertainties in $M_{{\mathrm {B}} ^{*}}^{\mathrm {PDG}}-M_{{\mathrm {B}}}^{\mathrm {PDG}}$	7.7	4.8	–	–
Finite size of simulated samples	1.1	1.3	1.1	1.3
Total	19	15	7.8	24

### Systematic uncertainties in the ratios $R^{\pm }_{2*}$, $R^{0}_{2*}$, $R^{\pm }_{\sigma }$, and $R^{0}_{\sigma }$

No systematic uncertainty related to the track reconstruction efficiency is assigned to the ratios considered in this subsection, since they involve final states in the numerator and denominator with equal numbers of charged particles.

In order to evaluate the systematic uncertainties related to the choice of the invariant mass fit model, several alternative functions are tested, as in the previous subsection. The systematic uncertainty in each ratio is calculated as the largest deviation of the corresponding ratio of signal yields obtained using alternative fit models with respect to the baseline fit model. The uncertainties related to the fits to ${\mathrm {B}} ^+ \pi ^-$, ${\mathrm {B}} ^+ \mathrm {K} ^-$, and ${\mathrm {B}} ^0 \mathrm {K} ^0_{\mathrm {S}}$ invariant mass distributions are treated separately and include:

- A systematic uncertainty related to the fit to ${\mathrm {B}} ^+ \pi ^-$ invariant mass of 2.9% for $N({\mathrm {B}} ^{*}_{{\mathrm {s}}2} \rightarrow {\mathrm {B}} ^{*+} \mathrm {K} ^- )/N({\mathrm {B}} ^{*}_{{\mathrm {s}}2} \rightarrow {\mathrm {B}} ^+ \mathrm {K} ^- )$ and 2.7% for $N({\mathrm {B}} _{{\mathrm {s}}1} \rightarrow {\mathrm {B}} ^{*+} \mathrm {K} ^- )/N({\mathrm {B}} ^{*}_{{\mathrm {s}}2} \rightarrow {\mathrm {B}} ^+ \mathrm {K} ^- )$,
- A systematic uncertainty related to the fit to ${\mathrm {B}} ^+ \mathrm {K} ^-$ invariant mass of 17% for $N({\mathrm {B}} ^{*}_{{\mathrm {s}}2} \rightarrow {\mathrm {B}} ^{*+} \mathrm {K} ^- )/N({\mathrm {B}} ^{*}_{{\mathrm {s}}2} \rightarrow {\mathrm {B}} ^+ \mathrm {K} ^- )$ and 7.1% for $N({\mathrm {B}} _{{\mathrm {s}}1} \rightarrow {\mathrm {B}} ^{*+} \mathrm {K} ^- )/N({\mathrm {B}} ^{*}_{{\mathrm {s}}2} \rightarrow {\mathrm {B}} ^+ \mathrm {K} ^- )$,
- A systematic uncertainty related to the fit to ${\mathrm {B}} ^0 \mathrm {K} ^0_{\mathrm {S}}$ invariant mass of 13% for $N({\mathrm {B}} ^{*}_{{\mathrm {s}}2} \rightarrow {\mathrm {B}} ^{*0} \mathrm {K} ^0_{\mathrm {S}} )/N({\mathrm {B}} ^{*}_{{\mathrm {s}}2} \rightarrow {\mathrm {B}} ^0 \mathrm {K} ^0_{\mathrm {S}} )$ and 24% for the ratio $N({\mathrm {B}} _{{\mathrm {s}}1} \rightarrow {\mathrm {B}} ^{*0} \mathrm {K} ^0_{\mathrm {S}} )/N({\mathrm {B}} ^{*}_{{\mathrm {s}}2} \rightarrow {\mathrm {B}} ^0 \mathrm {K} ^0_{\mathrm {S}} )$.

The systematic uncertainty in the ratios $$R^{\pm }_{2*} $$, $$R^{0}_{2*} $$, $$R^{\pm }_{\sigma } $$, and $$R^{0}_{\sigma } $$, related to the knowledge of the invariant mass resolution is estimated as in the previous subsection, and is found to be in the range 1.2–3.0%.

The systematic uncertainty associated with the uncertainty in the mass differences $M_{{\mathrm {B}} ^{*+}}^{\mathrm {PDG}}-M_{{\mathrm {B}} ^+}^{\mathrm {PDG}}$ and $M_{{\mathrm {B}} ^{*0}}^{\mathrm {PDG}}-M_{{\mathrm {B}} ^0}^{\mathrm {PDG}}$ must be taken into account, since these values are fixed in the fits. The baseline fits are repeated with each mass difference fixed to its nominal value plus and minus its uncertainty, and the largest deviations from the baseline of the obtained ratios of signal yields are taken as systematic uncertainties: 7.7% for $N({\mathrm {B}} ^{*}_{{\mathrm {s}}2} \rightarrow {\mathrm {B}} ^{*+} \mathrm {K} ^- )/N({\mathrm {B}} ^{*}_{{\mathrm {s}}2} \rightarrow {\mathrm {B}} ^+ \mathrm {K} ^- )$ and 4.8% for $N({\mathrm {B}} ^{*}_{{\mathrm {s}}2} \rightarrow {\mathrm {B}} ^{*0} \mathrm {K} ^0_{\mathrm {S}} )/N({\mathrm {B}} ^{*}_{{\mathrm {s}}2} \rightarrow {\mathrm {B}} ^0 \mathrm {K} ^0_{\mathrm {S}} )$. The changes in other ratios under variations of $M_{{\mathrm {B}} ^{*+}}^{\mathrm {PDG}}-M_{{\mathrm {B}} ^+}^{\mathrm {PDG}}$ and $M_{{\mathrm {B}} ^{*0}}^{\mathrm {PDG}}-M_{{\mathrm {B}} ^0}^{\mathrm {PDG}}$ are negligible.

The systematic uncertainties due to non-$$\mathrm {K} ^{*0} $$ contributions cancel out in the ratios $$R^{0}_{2*} $$ and $$R^{0}_{\sigma } $$.

Table 4 lists those systematic uncertainties, together with the total ones, calculated by summing the different contributions in quadrature.

### Systematic uncertainties in the mass differences and natural widths

The fits to the ${\mathrm {B}} \mathrm {K}$ invariant mass distributions are also used to measure the mass differences

$$\begin{aligned} {\begin{aligned} \varDelta M_{{\mathrm {B}} ^{*}_{{\mathrm {s}}2}}^{\pm }&= M({\mathrm {B}} ^{*}_{{\mathrm {s}}2})-M_{{\mathrm {B}} ^+}^{\mathrm {PDG}}-M_{\mathrm {K} ^-}^{\mathrm {PDG}}, \\ \varDelta M_{{\mathrm {B}} _{{\mathrm {s}}1}}^{\pm }&= M({\mathrm {B}} _{{\mathrm {s}}1})-M_{{\mathrm {B}} ^{*+}}^{\mathrm {PDG}}-M_{\mathrm {K} ^-}^{\mathrm {PDG}}, \\ \varDelta M_{{\mathrm {B}} ^{*}_{{\mathrm {s}}2}}^{0}&= M({\mathrm {B}} ^{*}_{{\mathrm {s}}2})-M_{{\mathrm {B}} ^0}^{\mathrm {PDG}}-M_{\mathrm {K} ^0_{\mathrm {S}}}^{\mathrm {PDG}}, \\ \varDelta M_{{\mathrm {B}} _{{\mathrm {s}}1}}^{0}&= M({\mathrm {B}} _{{\mathrm {s}}1})-M_{{\mathrm {B}} ^{*0}}^{\mathrm {PDG}}-M_{\mathrm {K} ^0_{\mathrm {S}}}^{\mathrm {PDG}}. \end{aligned} } \end{aligned}$$

Using these values, the mass differences

$$\begin{aligned} M_{{\mathrm {B}} ^0}-M_{{\mathrm {B}} ^+}&= \varDelta M_{{\mathrm {B}} ^{*}_{{\mathrm {s}}2}}^{\pm }-\varDelta M_{{\mathrm {B}} ^{*}_{{\mathrm {s}}2}}^{0} +M_{\mathrm {K} ^-}^{\mathrm {PDG}}-M_{\mathrm {K} ^0_{\mathrm {S}}}^{\mathrm {PDG}} \end{aligned}$$

and

$$\begin{aligned} M_{{\mathrm {B}} ^{*0}}-M_{{\mathrm {B}} ^{*+}}&= \varDelta M_{{\mathrm {B}} _{{\mathrm {s}}1}}^{\pm }-\varDelta M_{{\mathrm {B}} _{{\mathrm {s}}1}}^{0} +M_{\mathrm {K} ^-}^{\mathrm {PDG}}-M_{\mathrm {K} ^0_{\mathrm {S}}}^{\mathrm {PDG}} \end{aligned}$$

can be determined.

The natural width of the ${\mathrm {B}} ^{*}_{{\mathrm {s}}2}$ state is measured only in the ${\mathrm {B}} ^+ \mathrm {K} ^-$ channel due to the limited number of events in the ${\mathrm {B}} ^0 \mathrm {K} ^0_{\mathrm {S}}$ channel. Systematic uncertainties in these measurements are discussed in this subsection.

**Table 5:** Systematic uncertainties (in $\,\text {MeV}$) in the measured mass differences and natural width. The ${\mathrm {B}} ^{*}_{{\mathrm {s}}2}$ width is measured only in the ${\mathrm {B}} ^+ \mathrm {K} ^-$ channel

Source	$\varDelta M_{{\mathrm {B}} ^{*}_{{\mathrm {s}}2}}^{\pm }$	$\varDelta M_{{\mathrm {B}} _{{\mathrm {s}}1}}^{\pm }$	$\varDelta M_{{\mathrm {B}} ^{*}_{{\mathrm {s}}2}}^{0}$	$\varDelta M_{{\mathrm {B}} _{{\mathrm {s}}1}}^{0}$	$M_{{\mathrm {B}} ^0}-M_{{\mathrm {B}} ^+}$	$M_{{\mathrm {B}} ^{*0}}-M_{{\mathrm {B}} ^{*+}}$	$\varGamma _{{\mathrm {B}} ^{*}_{{\mathrm {s}}2}}$
$m_{{\mathrm {B}} ^+ \pi ^-}$ distribution model	0.024	0.008	–	–	0.024	0.008	0.11
$m_{{\mathrm {B}} ^+ \mathrm {K} ^-}$ distribution model	0.011	0.043	–	–	0.011	0.043	0.11
$m_{{\mathrm {B}} ^0 \mathrm {K} ^0_{\mathrm {S}}}$ distribution model	–	–	0.039	0.038	0.039	0.038	–
Uncertainties in $M_{{\mathrm {B}} ^{*}}^{\mathrm {PDG}}-M_{{\mathrm {B}}}^{\mathrm {PDG}}$	0.012	0.003	0.003	0.0001	0.012	0.003	0.03
Shift from reconstruction	0.056	0.044	0.050	0.042	0.075	0.061	–
Detector misalignment	0.036	0.005	0.031	0.006	0.038	0.008	0.15
Mass resolution	0.007	0.005	0.005	0.005	0.009	0.007	0.20
Total	0.073	0.063	0.071	0.057	0.098	0.085	0.30

The uncertainty related to the choice of the fit model is estimated by testing alternative fit models, as in Sect. 5.1. The largest deviation from the mass difference obtained from each baseline fit value is taken as the systematic uncertainty in the respective mass difference or natural width. The uncertainties related to the fits to the ${\mathrm {B}} ^+ \pi ^-$, ${\mathrm {B}} ^+ \mathrm {K} ^-$, and ${\mathrm {B}} ^0 \mathrm {K} ^0_{\mathrm {S}}$ invariant mass distributions are treated separately.

The systematic uncertainty associated with the knowledge of the mass difference $M_{{\mathrm {B}} ^{*+}}^{\mathrm {PDG}}-M_{{\mathrm {B}} ^+}^{\mathrm {PDG}}$ (or $M_{{\mathrm {B}} ^{*0}}^{\mathrm {PDG}}-M_{{\mathrm {B}} ^0}^{\mathrm {PDG}}$) is taken into account as well: the baseline fits are repeated with the mass difference $M_{{\mathrm {B}} ^{*}}^{\mathrm {PDG}}-M_{{\mathrm {B}}}^{\mathrm {PDG}}$ fixed to its nominal value plus or minus its uncertainty. The largest deviation from the baseline of the obtained mass differences and natural width is taken as the corresponding systematic uncertainty.

Studies of simulated events show that the mass differences measured in the reconstructed invariant mass distributions are slightly shifted with respect to the mass differences used in the generation of simulated events. Therefore, the measured mass differences are corrected by the observed shifts (which are up to 0.056$\,\text {MeV}$), and each shift is conservatively treated as a systematic uncertainty in the respective mass-difference measurement.

In order to estimate the systematic uncertainties due to possible misalignment of the detector [21], eighteen different simulated samples with various distorted geometries are produced and analyzed for each of the four decay channels. From these measurements the largest deviation of the estimation of the invariant mass or its resolution from the perfectly aligned case is accepted as an estimate of the systematic uncertainty from a possible detector misalignment. The magnitudes of distortions are large enough to be detected and corrected by the standard alignment procedures [21]. The shifts in the measured mass differences observed in these simulations are up to 0.038$\,\text {MeV}$. The systematic uncertainty in the invariant mass resolution of the ${\mathrm {B}} ^{*}_{{\mathrm {s}}2} \rightarrow {\mathrm {B}} ^+ \mathrm {K} ^-$ signal is found to be 0.042$\,\text {MeV}$, and the corresponding uncertainty in $\varGamma _{{\mathrm {B}} ^{*}_{{\mathrm {s}}2}}$ is obtained by repeating the baseline fit with the resolution increased or decreased by this value. The largest deviation in the measured natural width with respect to the baseline value is used as a systematic uncertainty.

The systematic uncertainties related to the invariant mass resolution are estimated in the same way as in the previous subsections and are found to be up to 0.007$\,\text {MeV}$ for the mass differences and 0.2$\,\text {MeV}$ for the natural width. This source of uncertainty is conservatively considered to be uncorrelated with the systematic uncertainty related to a possible detector misalignment.

These systematic uncertainties are summarized in Table 5, together with the total systematic uncertainties, calculated by summing in quadrature the different contributions. It was checked that the mass of the ${\mathrm {B}} ^+$ meson, measured in the ${\mathrm {B}} ^+ \rightarrow {\mathrm {J}}/\psi \mathrm {K} ^+$ decay, is consistent with the world-average value, after taking into account the systematic uncertainties related to the shift from the reconstruction and possible detector misalignment.

## Results

The decay ${\mathrm {B}} ^{*}_{{\mathrm {s}}2} \rightarrow {\mathrm {B}} ^0 \mathrm {K} ^0_{\mathrm {S}}$ is observed for the first time with a corresponding statistical significance of 6.3 standard deviations. The first evidence (3.9 standard deviations) for the decay ${\mathrm {B}} _{{\mathrm {s}}1} \rightarrow {\mathrm {B}} ^{*0} \mathrm {K} ^0_{\mathrm {S}}$ is found. In the measurements presented below of the relative branching fractions, cross sections multiplied by branching fractions, masses, mass differences, and natural width, the first uncertainty is statistical, the second is systematic, and if there is a third, it is related to the uncertainties in the world-average values of the branching fractions, masses, and mass differences [12].

Formulae (1)–(4) are used with the branching fractions [12] $\mathcal {B}({\mathrm {B}} ^+ \rightarrow {\mathrm {J}}/\psi \mathrm {K} ^+ )=(1.026\pm 0.031)\,10^{-3},$ $\mathcal {B}({\mathrm {B}} ^0 \rightarrow {\mathrm {J}}/\psi \mathrm {K} ^{*0} )=(1.28\pm 0.05)\,10^{-3},$ $\mathcal {B}(\mathrm {K} ^{*0} \rightarrow \mathrm {K} ^+ \pi ^- )=(0.99754\pm 0.00021),$ and $\mathcal {B}(\mathrm {K} ^0_{\mathrm {S}} \rightarrow \pi ^+\pi ^- )=(0.6920\pm 0.0005)$ to determine the following ratios of branching fractions:

$$\begin{aligned} R^{0\pm }_{2}&= \frac{\mathcal {B}({\mathrm {B}} ^{*}_{{\mathrm {s}}2} \rightarrow {\mathrm {B}} ^0 \mathrm {K} ^0_{\mathrm {S}} )}{\mathcal {B}({\mathrm {B}} ^{*}_{{\mathrm {s}}2} \rightarrow {\mathrm {B}} ^+ \mathrm {K} ^- )} = 0.432\pm 0.077\pm 0.075\pm 0.021, \\ R^{0\pm }_{1}&= \frac{\mathcal {B}({\mathrm {B}} _{{\mathrm {s}}1} \rightarrow {\mathrm {B}} ^{*0} \mathrm {K} ^0_{\mathrm {S}} )}{\mathcal {B}({\mathrm {B}} _{{\mathrm {s}}1} \rightarrow {\mathrm {B}} ^{*+} \mathrm {K} ^- )} = 0.49\pm 0.12\pm 0.07\pm 0.02, \\ R^{\pm }_{2*}&= \frac{\mathcal {B}({\mathrm {B}} ^{*}_{{\mathrm {s}}2} \rightarrow {\mathrm {B}} ^{*+} \mathrm {K} ^- )}{\mathcal {B}({\mathrm {B}} ^{*}_{{\mathrm {s}}2} \rightarrow {\mathrm {B}} ^+ \mathrm {K} ^- )} = 0.081\pm 0.021\pm 0.015, \\ R^{0}_{2*}&= \frac{\mathcal {B}({\mathrm {B}} ^{*}_{{\mathrm {s}}2} \rightarrow {\mathrm {B}} ^{*0} \mathrm {K} ^0_{\mathrm {S}} )}{\mathcal {B}({\mathrm {B}} ^{*}_{{\mathrm {s}}2} \rightarrow {\mathrm {B}} ^0 \mathrm {K} ^0_{\mathrm {S}} )} = 0.093\pm 0.086\pm 0.014. \end{aligned}$$

The ratio $R^{0\pm }_{2}$ is in good agreement with the theoretical predictions of about 0.43 [22, 23], while the ratio $R^{0\pm }_{1}$ is 2.5 standard deviations away from the theoretical prediction of 0.23 [22], which, however, has no uncertainty estimate. The third ratio is in agreement with the measurements of LHCb [5] and CDF [6]: $0.093\pm 0.013\pm 0.012$ and $0.10\pm 0.03\pm 0.02$, respectively. It is also consistent with the theoretical predictions [22–25]. The fourth ratio is a new result.

In addition, using Eqs. (5)–(6), the ratios of production cross sections times branching fractions are measured:

$$\begin{aligned} {\begin{aligned} R^{\pm }_{\sigma }&= \frac{\sigma (\mathrm {pp} \rightarrow {\mathrm {B}} _{{\mathrm {s}}1} \mathrm {X})\,\mathcal {B}({\mathrm {B}} _{{\mathrm {s}}1} \rightarrow {\mathrm {B}} ^{*+} \mathrm {K} ^- )}{\sigma (\mathrm {pp} \rightarrow {\mathrm {B}} ^{*}_{{\mathrm {s}}2} \mathrm {X})\,\mathcal {B}({\mathrm {B}} ^{*}_{{\mathrm {s}}2} \rightarrow {\mathrm {B}} ^+ \mathrm {K} ^- )} \\&= 0.233\pm 0.019\pm 0.018, \\ R^{0}_{\sigma }&= \frac{\sigma (\mathrm {pp} \rightarrow {\mathrm {B}} _{{\mathrm {s}}1} \mathrm {X})\,\mathcal {B}({\mathrm {B}} _{{\mathrm {s}}1} \rightarrow {\mathrm {B}} ^{*0} \mathrm {K} ^0_{\mathrm {S}} )}{\sigma (\mathrm {pp} \rightarrow {\mathrm {B}} ^{*}_{{\mathrm {s}}2} \mathrm {X})\,\mathcal {B}({\mathrm {B}} ^{*}_{{\mathrm {s}}2} \rightarrow {\mathrm {B}} ^0 \mathrm {K} ^0_{\mathrm {S}} )} \\&= 0.266\pm 0.079\pm 0.063. \end{aligned}} \end{aligned}$$

The value of $R^{\pm }_{\sigma }$ was previously determined by LHCb to be $0.232\pm 0.014\pm 0.013$ [5] at $\sqrt{s}=7\,\text {TeV}$ and in a different pseudorapidity region, consistent with the result presented here.

The following mass differences are obtained:

$$\begin{aligned} {\begin{aligned} \varDelta M_{{\mathrm {B}} ^{*}_{{\mathrm {s}}2}}^{\pm }&= M({\mathrm {B}} ^{*}_{{\mathrm {s}}2})-M_{{\mathrm {B}} ^+}^{\mathrm {PDG}}-M_{\mathrm {K} ^-}^{\mathrm {PDG}} \\&= 66.87\pm 0.09\pm 0.07\,\text {MeV}, \\ \varDelta M_{{\mathrm {B}} ^{*}_{{\mathrm {s}}2}}^{0}&= M({\mathrm {B}} ^{*}_{{\mathrm {s}}2})-M_{{\mathrm {B}} ^0}^{\mathrm {PDG}}-M_{\mathrm {K} ^0_{\mathrm {S}}}^{\mathrm {PDG}} \\&= 62.37\pm 0.48\pm 0.07\,\text {MeV}, \\ \varDelta M_{{\mathrm {B}} _{{\mathrm {s}}1}}^{\pm }&= M({\mathrm {B}} _{{\mathrm {s}}1})-M_{{\mathrm {B}} ^{*+}}^{\mathrm {PDG}}-M_{\mathrm {K} ^-}^{\mathrm {PDG}} \\&= 10.45\pm 0.09\pm 0.06\,\text {MeV}, \\ \varDelta M_{{\mathrm {B}} _{{\mathrm {s}}1}}^{0}&= M({\mathrm {B}} _{{\mathrm {s}}1})-M_{{\mathrm {B}} ^{*0}}^{\mathrm {PDG}}-M_{\mathrm {K} ^0_{\mathrm {S}}}^{\mathrm {PDG}} \\&= 5.61\pm 0.23\pm 0.06\,\text {MeV}. \end{aligned}} \end{aligned}$$

The first two mass differences are in good agreement with LHCb [5] and CDF [6] results (see Table 1). Using these two measurements, the world-average masses of the ${\mathrm {B}} ^+$ and $\mathrm {K} ^-$ mesons, and the mass difference $M_{{\mathrm {B}} ^{*+}}^{\mathrm {PDG}}-M_{{\mathrm {B}} ^+}^{\mathrm {PDG}}$, the ${\mathrm {B}} ^{(*)}_{{\mathrm {s}}1,2}$ masses are determined:

$$\begin{aligned}\begin{aligned} M({\mathrm {B}} ^{*}_{{\mathrm {s}}2})&= 5839.86\pm 0.09\pm 0.07\pm 0.15\,\text {MeV},\\ M({\mathrm {B}} _{{\mathrm {s}}1})&= 5828.78\pm 0.09\pm 0.06\pm 0.28\,\text {MeV}.\\ \end{aligned}\end{aligned}$$

The measured masses in the ${\mathrm {B}} ^0 \mathrm {K} ^0_{\mathrm {S}}$ channel are consistent with our results using the ${\mathrm {B}} ^+ \mathrm {K} ^-$ channel but have significantly larger uncertainties.

Using the mass-difference measurements above, the mass differences between the neutral and charged ${\mathrm {B}}$ and ${\mathrm {B}} ^{*}$ mesons are found to be:

$$\begin{aligned}\begin{aligned} M_{{\mathrm {B}} ^0}-M_{{\mathrm {B}} ^+}&= 0.57\pm 0.49\pm 0.10\pm 0.02\,\text {MeV}, \\ M_{{\mathrm {B}} ^{*0}}-M_{{\mathrm {B}} ^{*+}}&= 0.91\pm 0.24\pm 0.09\pm 0.02\,\text {MeV}. \end{aligned}\end{aligned}$$

The first mass difference result is consistent with the significantly more precise world-average value of $0.31\pm 0.06\,\text {MeV}$ [12]. There are no previous measurements of $M_{{\mathrm {B}} ^{*0}}-M_{{\mathrm {B}} ^{*+}}$, and this paper presents a new method to measure both of these mass differences.

Lastly, the natural width of the ${\mathrm {B}} ^{*}_{{\mathrm {s}}2}$ meson is determined to be

$$\begin{aligned} \varGamma _{{\mathrm {B}} ^{*}_{{\mathrm {s}}2}} = 1.52\pm 0.34\pm 0.30\,\text {MeV}, \end{aligned}$$

consistent with the results of LHCb [5] and CDF [6] (see Table 1).

## Summary

The *P*-wave ${\mathrm {B}} ^0_{\mathrm {s}}$ meson states are studied using a data sample corresponding to an integrated luminosity of  of proton-proton collisions collected by the CMS experiment at $\sqrt{s}= 8\,\text {TeV}$ in 2012. Observation and evidence are reported for the decays ${\mathrm {B}} ^{*}_{{\mathrm {s}}2}(5840)^0 \rightarrow {\mathrm {B}} ^0 \mathrm {K} ^0_{\mathrm {S}}$ and ${\mathrm {B}} _{{\mathrm {s}}1}(5830)^0 \rightarrow {\mathrm {B}} ^{*0} \mathrm {K} ^0_{\mathrm {S}}$, respectively. Four ratios of branching fractions and two ratios of production cross sections multiplied by the branching fractions of the *P*-wave ${\mathrm {B}} ^0_{\mathrm {s}}$ mesons into a ${\mathrm {B}}$ meson and kaon are measured. In addition, the differences between the ${\mathrm {B}} ^{(*)}_{{\mathrm {s}}1,2}$ mass and the sum of the ${\mathrm {B}}$ meson and kaon mass are determined, as well as the ${\mathrm {B}} ^{*}_{{\mathrm {s}}2}(5840)^0$ natural width. Finally, using a new approach, the mass differences $M_{{\mathrm {B}} ^0}-M_{{\mathrm {B}} ^+}$ and $M_{{\mathrm {B}} ^{*0}}-M_{{\mathrm {B}} ^{*+}}$ are measured, where the latter is determined for the first time.
